# Dataset of tensile strength development of concrete with manufactured sand

**DOI:** 10.1016/j.dib.2017.02.043

**Published:** 2017-02-22

**Authors:** Shunbo Zhao, Feijia Hu, Xinxin Ding, Mingshuang Zhao, Changyong Li, Songwei Pei

**Affiliations:** a School of Civil Engineering and Communication, North China University of Water Resources and Electric Power, No. 136 Jinshui East Road, 450046 Zhengzhou, China; b Henan Province International United Lab of Eco-building Materials and Engineering, No. 36 Beihuan Road, 450045 Zhengzhou, China

**Keywords:** Concrete with manufactured sand (MSC), Stone powder, Water-cement ratio, Splitting tensile strength

## Abstract

This article presents 755 groups splitting tensile strength tests data of concrete with manufactured sand (MSC) in different curing age ranged from 1 day to 388 days related to the research article “Experimental study on tensile strength development of concrete with manufactured sand” (Zhao et al., 2017) [1]. These data were used to evaluate the precision of the prediction formulas of tensile strength of MSC, and can be applied as dataset for further studies.

**Specifications Table**

**Table:** 

Subject area	Construction and building materials
More specific subject area	Building materials
Type of data	Table
How data was acquired	Tests and collection
Data format	Raw and filtered
Experimental factors	Publicly available data sources
Experimental features	Testing the splitting tensile strengths at different curing age of MSC with different stone powder content and water-cement ratio (or water-binder ratio) in laboratory situation.
Data source location	Zhengzhou City, China.
Data accessibility	Data within this article.
Related research article	S.B Zhao, X.X Ding, M.S Zhao, C.Y. Li, S.W Pei. Experimental study on tensile strength development of concrete with manufactured sand. Constr. Build. Mater. In press.

**Value of the data**

• The data indicating splitting tensile strength of MSC at different curing age in laboratory situation.
• The data are publicly available, but are scattered in many different articles.
• Be useful for comparing tensile strength of MSC with that of concrete made by different aggregates.

## 1 Data

755 groups splitting tensile strength test data of MSC were assembled from 41 experimental studies [1], [2], [3], [4], [5], [6], [7], [8], [9], [10], [11], [12], [13], [14], [15], [16], [17], [18], [19], [20], [21], [22], [23], [24], [25], [26], [27], [28], [29], [30], [31], [32], [33], [34], [35], [36], [37], [38], [39], [40], [41] including detailed properties of raw materials and mix proportions as well as basic properties of fresh and hardened MSC, which were collected from authors’ experiments and other researches presented.

## 2 Experimental design, materials and methods

Table A gives 755 groups splitting tensile strength tests data of MSC in different curing age ranged from 1 day to 388 days. Raw materials of MSC were the ordinary silicate cements, the admixture consisted of fly ash, slag and silica fume, the crushed stone and the manufactured sand. The cement’ compressive strength and tensile strength at 28 days ranged in 35.5–63.4 MPa and 6.9–10.8 MPa, respectively. The maximum grain size of crushed stone ranged from 12 mm to 120 mm. The fineness modulus of manufactured sand was 2.2–3.55. As these studies were done based on different codes, different maximum particle sizes of 0.075 mm and 0.160 mm were defined for stone powder in manufactured sand. The contents of stone powder with particle size of 0–0.075 mm ranged in 0–21.8%, whereas those with particle size of 0~0.160 mm varied in 0~40%. The water-binder ratio *W*/*B*=0.24–1.00, while the water-cement ratio *m*_w_/*m*_c_=0.30~1.43. The sand ratio was 24–54%. The compressive strength of MSC at 28 days ranged from 10.1–96.3 MPa, the slump of fresh MSC varied from 10 mm to 260 mm, the curing time of specimens ranged from 1 day to 388 days.

## References

[1] S.B. Zhao, X.X. Ding, M.S. Zhao, C.Y. Li, K.Q. Yao, Experimental study on tensile strength development of concrete with manufactured sand, Constr. Build. Mater., 2017, In press.

[2] Li F.L., Liu C.J., Pan L.Y., Li C.Y. (2013). Machine-Made Sand Concrete.

[3] Chai S.H. (2014). Experimental Study and Mechanism Analysis on Mechanical Property of High Performance Concrete with Manufactured Sand After High Temperature (Thesis for Master Degree).

[4] He S.D., Li L., Li G.H., Wang H. (2014). Experimental study on split strength of manufactured sand concrete. Concrete.

[5] He S.D., Liu L.X., Wang H., Wang R.L. (2010). Experimental research on compressive strength of manufactured sand concrete in different replacement ratios. Concrete.

[6] Jiang F.F. (2012). Research of High Performance Manufactured Sand Concrete (Thesis for Master Degree).

[7] Li L., Li H.D., Zhang J.H. (2015). Experimental study on and application of artificial sand used for marine engineering concrete in Northern China. Port. Waterw. Eng..

[8] Li Z.C., Zhang A.J. (2015). The positive and negative effects of manufactured sand on shrinkage and strength of concrete. Highway.

[9] Shi W.T. (2010). Quarry Reject Stone Chips in Concretes Application.

[10] Wang L.Y., Xu L.W. (2014). Orthogonal experimental research on the strength and workability of machine-made sand concrete. J. Shandong Jianzhu Univ..

[11] Yang Y.H. (2007). Study on Preparation and Properties of the C80 Manufactured Sand Concrete (Thesis for Master Degree).

[12] Zhang R.H. (2008). Experimental Study on the Property of Self-Compacting Concrete (SCC) by Stone Chips.

[13] Lu Y.J. (2016). The test of the influence of dust content of crushed limestone aggregate on concrete performance. Yunnan Water Power.

[14] Li X.Y., Xie G.S., Ren J.M., Xing J.Q. (2016). Effect of mica-rich stone powder contained in manufactured sand on the properties of abrasion resistance concrete. Water Power.

[15] Chen H.Y. (2015). Study on the performance of high performance concrete prepared by the northeast of Yunnan. Sci. Technol. West China.

[16] Lin L.K. (2015). Research of High Performance Manufactured Sand Concrete.

[17] Lin Y.Z. (2014). Study on Application of C55 Machine-Made Sand in Bridge Girder.

[18] Wang W. (2014). Preparation of High Performance Concrete with Iron Tailings and Barron Rocks as Aggregates.

[19] Yang X.L. (2013). Tailings Aggregate Green High Performance Concrete with Mix Design and Application Research.

[20] He H.W., Yao Q. (2006). Study on the flowing and high strength manufactured sand concrete. Chongqing Arch..

[21] Wang J.L. (2008). Research of Effect Sand Mechanism of Manufacture Sand Characteristics on Portland Cement Concrete.

[22] Cao Z.L., Yang Y.H. (2010). Produce C60 self-compacting steel-pipe concrete with machine-made sand. Concrete.

[23] Wu M.W., Fu Z.G., Li T.X., Wang D.H. (2000). Experimental Study on the effects of stone power content on properties of concrete. Railw. Constr. Technol..

[24] Li X. (2005). Comparative analysis of mechanical properties of concretes mixed with Hubo machine-made sand and river sand. Shanxi Arch..

[25] Tan K.Z. (1998). Experimental study on Stone powder content limit and using in Huangdan hydropower station. Sichuan Water Power.

[26] Huang X.T., Han Z.J. (1995). Research on effect of artificial rock ash content on concrete properties. Water Power.

[27] Cai J.W. (2006). Research of Effects and Mechanism of Micro Fines on Manufactured Fine Aggregate Concretes.

[28] Liu J., Wang L.H., Zhan Z.F., Lei D. (2013). Study on the influence of artificial sand on the comprehensive performance of concrete. Guangdong Water Resour. Hydropower.

[29] Qin G.Y. (2008). Study on Manufactured Sand Fair-faced Concrete and Its Apparent Quality Evolution.

[30] Wang Z.H., Chen R.H. (1998). Experimental study on mechanical properties of fully graded aggregate concrete in TGP. J. Yangtze River Sci. Res. Inst..

[31] Chen Z.W. (2001). Test and study on the properties of artificial-sand concrete with high content of stone powder for Mianhuatan Hydropower Station. Water Power.

[32] Wang Y., Lin L.X., Yi Z.F. (2007). The influence of artificial granulated substance powder on performance of concrete. Concrete.

[33] Xing J.Q., Zhan S.L., Li X.Y., Xie G.S. (2015). Study on measuring method of mica content in manufactured sand powder and its effect on performance of concrete. New Build. Mater..

[34] Hong J.X., Jiang L.H., Huang W., Miao C.W., Ye Y.Q. (2005). Research on the effect of stone powder contained in artificial sand on the concrete performance and its acting mechanism. J. Highw. Transp. Res. Dev..

[35] Li J.Z., Ju G.D. (2001). Study on properties of concrete prepared with stone sands of Gushuling in Three Gorges Project. J. Yangtze River Sci. Res. Inst..

[36] J.Z. Li, Y.C. Wang, J.E. Yan, H.Q. Yang, Study on the application of stone sand in concrete of the Three Gorges Project, China Materials Conference (in Chinese), 2002.

[37] Fu W.Y. (2002). Research and application of the performance of the concrete with stone sand in the Three Gorges Project. Yunnan Water Power.

[38] Q.H. Yang, J.Z. Li, W.J. Dong, L.L. Tan, Mix design and performance test of roller compacted concrete in cofferdam of the Three Gorges Project, The technology of conference of 200 m grade of roller compacted concrete dam(in Chinese), 2006.

[39] Li X.Y., Ren J.M., Chen Y.H. (2012). Study on performances of concrete with high content of mica in stone powder. J. Hydroelectr. Eng..

[40] Xing J.Q. (2015). Study on Effect of Free Mica in Manufactured Sand Powder on Performance of Cement Mortar and Concrete.

[41] Parra C., Valcuende M., Gómez F. (2011). Splitting tensile strength and modulus of elasticity of self-compacting concrete. Const. Build. Mater..

