# Supplementary figures and images for: Dataset of tensile strength development of concrete with manufactured sand
Source: Data Brief. 2017 Feb 22;11:469–72. doi: 10.1016/j.dib.2017.02.043 (PMC5338904; doi:10.1016/j.dib.2017.02.043)

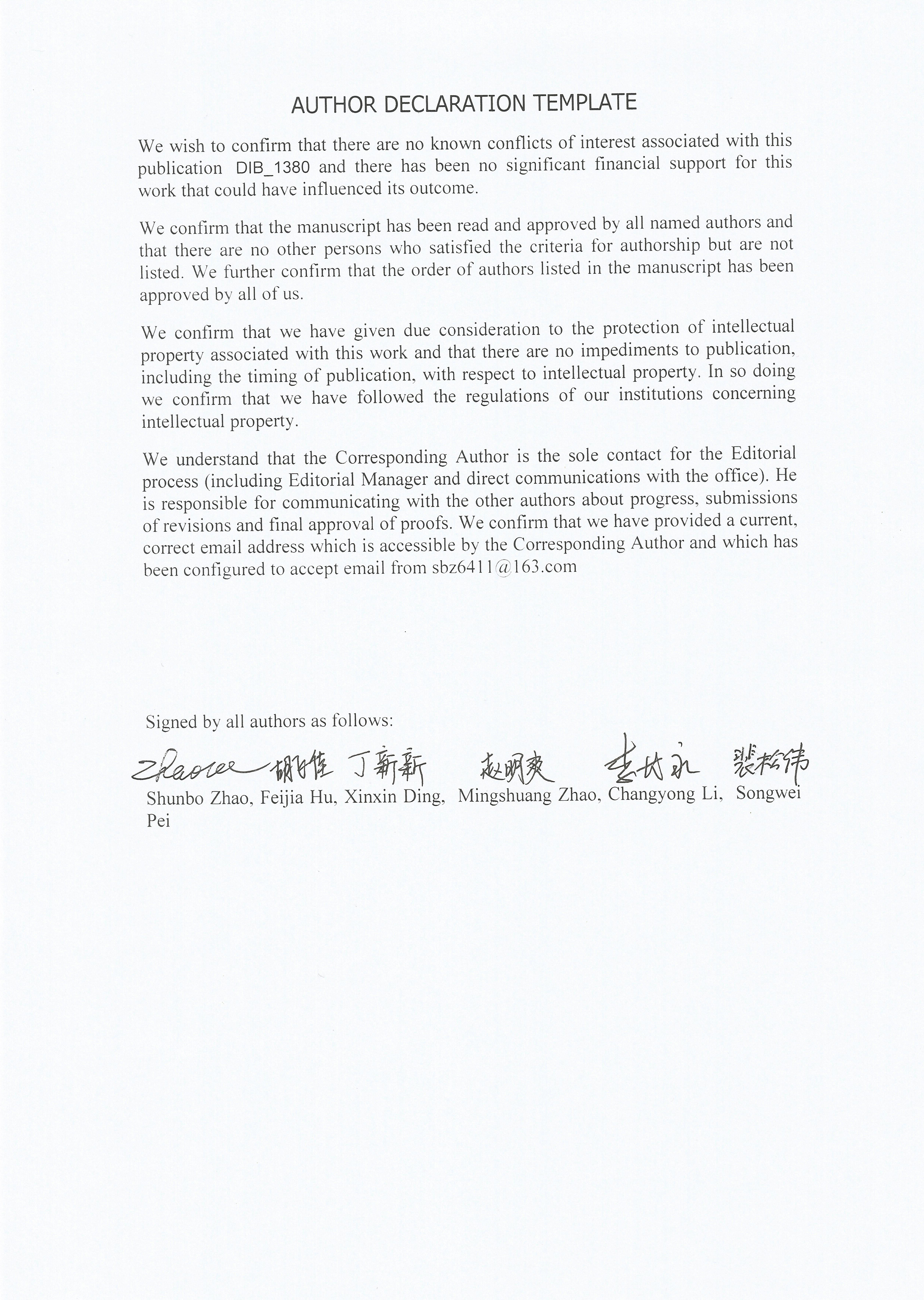

Supplement: Supplementary file 1 — Supplementary material [file mmc1.jpg]
