# Supplementary material for: Dataset of tensile strength development of concrete with manufactured sand
Source: Data Brief. 2017 Feb 22;11:469–72. doi: 10.1016/j.dib.2017.02.043 (PMC5338904; doi:10.1016/j.dib.2017.02.043)
Supplement: Supplementary file 2 — Supplementary material [file mmc2.docx]

**Supplementary Data**

Test database of tensile strength and compressive strength at *t* days of concrete with machine-made sand

Test database of concrete with machine-made sand at *t* days

| Source | Compressive  strength of  cement  *f*_ce_(MPa) | Tensile strength of cement  *f*_ct_(MPa) | Curing  age  (day) | *D*_max_ of  Crushed  stone  (mm) | Stone powder content in  Sand (%) | Fineness  modulus  of sand | W/B | Water to cement ratio  *m*_w_*/m*_c_ | Water  (kg/m^3^) | Sand ratio  (%) | Slump  (mm) | Compressive strength  *f*_cu,t_  (MPa) | Splitting tensile  strength  *f*_st,t_(MPa) |
| --- | --- | --- | --- | --- | --- | --- | --- | --- | --- | --- | --- | --- | --- |
| Zhao et al [1] | 46.8 | 8.0 | 3 | 31.5 | 5 | 3.34 | 0.56 | 0.56 | 180 | 44 | 50 | 32.5 | 1.18 |
| Zhao et al [1] | 46.8 | 8.0 | 3 | 31.5 | 9 | 3.27 | 0.56 | 0.56 | 180 | 44 | 70 | 28.7 | 1.13 |
| Zhao et al [1] | 46.8 | 8.0 | 3 | 31.5 | 13 | 2.77 | 0.56 | 0.56 | 180 | 44 | 50 | 28.5 | 1.56 |
| Zhao et al [1] | 46.8 | 8.0 | 7 | 31.5 | 5 | 3.34 | 0.56 | 0.56 | 180 | 44 | 50 | 33.5 | 1.39 |
| Zhao et al [1] | 46.8 | 8.0 | 7 | 31.5 | 9 | 3.27 | 0.56 | 0.56 | 180 | 44 | 70 | 34.9 | 1.38 |
| Zhao et al [1] | 46.8 | 8.0 | 7 | 31.5 | 13 | 2.77 | 0.56 | 0.56 | 180 | 44 | 50 | 34.7 | 1.42 |
| Zhao et al [1] | 46.8 | 8.0 | 14 | 31.5 | 5 | 3.34 | 0.56 | 0.56 | 180 | 44 | 50 | 39.7 | 2.62 |
| Zhao et al [1] | 46.8 | 8.0 | 14 | 31.5 | 9 | 3.27 | 0.56 | 0.56 | 180 | 44 | 70 | 38.9 | 3.05 |
| Zhao et al [1] | 46.8 | 8.0 | 14 | 31.5 | 13 | 2.77 | 0.56 | 0.56 | 180 | 44 | 50 | 37.0 | 2.31 |
| Zhao et al [1] | 46.8 | 8.0 | 28 | 31.5 | 5 | 3.34 | 0.56 | 0.56 | 180 | 44 | 50 | 41.2 | 3.02 |
| Zhao et al [1] | 46.8 | 8.0 | 28 | 31.5 | 9 | 3.27 | 0.56 | 0.56 | 180 | 44 | 70 | 41.3 | 3.75 |
| Zhao et al [1] | 46.8 | 8.0 | 28 | 31.5 | 13 | 2.77 | 0.56 | 0.56 | 180 | 44 | 50 | 40.5 | 3.01 |
| Zhao et al [1] | 46.8 | 8.0 | 42 | 31.5 | 5 | 3.34 | 0.56 | 0.56 | 180 | 44 | 50 | 42.0 | 3.31 |
| Zhao et al [1] | 46.8 | 8.0 | 42 | 31.5 | 9 | 3.27 | 0.56 | 0.56 | 180 | 44 | 70 | 41.8 | 3.82 |
| Zhao et al [1] | 46.8 | 8.0 | 42 | 31.5 | 13 | 2.77 | 0.56 | 0.56 | 180 | 44 | 50 | 42.3 | 3.43 |
| Zhao et al [1] | 46.8 | 8.0 | 56 | 31.5 | 5 | 3.34 | 0.56 | 0.56 | 180 | 44 | 50 | 43.8 | 3.73 |
| Zhao et al [1] | 46.8 | 8.0 | 56 | 31.5 | 9 | 3.27 | 0.56 | 0.56 | 180 | 44 | 70 | 42.4 | 3.99 |
| Zhao et al [1] | 46.8 | 8.0 | 56 | 31.5 | 13 | 2.77 | 0.56 | 0.56 | 180 | 44 | 50 | 45.0 | 3.68 |
| Zhao et al [1] | 46.8 | 8.0 | 84 | 31.5 | 5 | 3.34 | 0.56 | 0.56 | 180 | 44 | 50 | 47.4 | 4.42 |
| Zhao et al [1] | 46.8 | 8.0 | 84 | 31.5 | 9 | 3.27 | 0.56 | 0.56 | 180 | 44 | 70 | 48.8 | 4.21 |
| Zhao et al [1] | 46.8 | 8.0 | 84 | 31.5 | 13 | 2.77 | 0.56 | 0.56 | 180 | 44 | 50 | 48.0 | 4.48 |
| Zhao et al [1] | 46.8 | 8.0 | 118 | 31.5 | 5 | 3.34 | 0.56 | 0.56 | 180 | 44 | 50 | 48.9 | 4.37 |
| Zhao et al [1] | 46.8 | 8.0 | 118 | 31.5 | 9 | 3.27 | 0.56 | 0.56 | 180 | 44 | 70 | 49.3 | 4.03 |
| Zhao et al [1] | 46.8 | 8.0 | 118 | 31.5 | 13 | 2.77 | 0.56 | 0.56 | 180 | 44 | 50 | 48.7 | 4.41 |
| Zhao et al [1] | 46.8 | 8.0 | 178 | 31.5 | 5 | 3.34 | 0.56 | 0.56 | 180 | 44 | 50 | 49.7 | 4.68 |
| Zhao et al [1] | 46.8 | 8.0 | 178 | 31.5 | 9 | 3.27 | 0.56 | 0.56 | 180 | 44 | 70 | 51.9 | 4.22 |
| Zhao et al [1] | 46.8 | 8.0 | 178 | 31.5 | 13 | 2.77 | 0.56 | 0.56 | 180 | 44 | 50 | 49.9 | 4.42 |
| Zhao et al [1] | 46.8 | 8.0 | 238 | 31.5 | 5 | 3.34 | 0.56 | 0.56 | 180 | 44 | 50 | 51.1 | 4.22 |
| Zhao et al [1] | 46.8 | 8.0 | 238 | 31.5 | 9 | 3.27 | 0.56 | 0.56 | 180 | 44 | 70 | 55.4 | 3.92 |
| Zhao et al [1] | 46.8 | 8.0 | 238 | 31.5 | 13 | 2.77 | 0.56 | 0.56 | 180 | 44 | 50 | 54.5 | 4.12 |
| Zhao et al [1] | 46.8 | 8.0 | 298 | 31.5 | 5 | 3.34 | 0.56 | 0.56 | 180 | 44 | 50 | 51.2 | 4.36 |
| Zhao et al [1] | 46.8 | 8.0 | 298 | 31.5 | 9 | 3.27 | 0.56 | 0.56 | 180 | 44 | 70 | 56.2 | 4.05 |
| Zhao et al [1] | 46.8 | 8.0 | 298 | 31.5 | 13 | 2.77 | 0.56 | 0.56 | 180 | 44 | 50 | 54.8 | 4.23 |
| Zhao et al [1] | 46.8 | 8.0 | 358 | 31.5 | 5 | 3.34 | 0.56 | 0.56 | 180 | 44 | 50 | 51.6 | 4.41 |
| Zhao et al [1] | 46.8 | 8.0 | 358 | 31.5 | 9 | 3.27 | 0.56 | 0.56 | 180 | 44 | 70 | 56.4 | 4.22 |
| Zhao et al [1] | 46.8 | 8.0 | 358 | 31.5 | 13 | 2.77 | 0.56 | 0.56 | 180 | 44 | 50 | 55.1 | 4.32 |
| Zhao et al [1] | 46.8 | 8.0 | 3 | 31.5 | 5 | 3.34 | 0.45 | 0.45 | 180 | 36 | 110 | 38.4 | 2.67 |
| Zhao et al [1] | 46.8 | 8.0 | 3 | 31.5 | 9 | 3.27 | 0.45 | 0.45 | 180 | 36 | 160 | 38.0 | 2.61 |
| Zhao et al [1] | 46.8 | 8.0 | 3 | 31.5 | 13 | 2.77 | 0.45 | 0.45 | 180 | 36 | 45 | 38.1 | 2.71 |
| Zhao et al [1] | 46.8 | 8.0 | 7 | 31.5 | 5 | 3.34 | 0.45 | 0.45 | 180 | 36 | 110 | 42.2 | 2.81 |
| Zhao et al [1] | 46.8 | 8.0 | 7 | 31.5 | 9 | 3.27 | 0.45 | 0.45 | 180 | 36 | 160 | 42.3 | 3.02 |
| Zhao et al [1] | 46.8 | 8.0 | 7 | 31.5 | 13 | 2.77 | 0.45 | 0.45 | 180 | 36 | 45 | 41.7 | 2.95 |
| Zhao et al [1] | 46.8 | 8.0 | 14 | 31.5 | 5 | 3.34 | 0.45 | 0.45 | 180 | 36 | 110 | 44.9 | 3.06 |
| Zhao et al [1] | 46.8 | 8.0 | 14 | 31.5 | 9 | 3.27 | 0.45 | 0.45 | 180 | 36 | 160 | 44.8 | 3.05 |
| Zhao et al [1] | 46.8 | 8.0 | 14 | 31.5 | 13 | 2.77 | 0.45 | 0.45 | 180 | 36 | 45 | 44.2 | 3.01 |
| Zhao et al [1] | 46.8 | 8.0 | 28 | 31.5 | 5 | 3.34 | 0.45 | 0.45 | 180 | 36 | 110 | 54.2 | 3.46 |
| Zhao et al [1] | 46.8 | 8.0 | 28 | 31.5 | 9 | 3.27 | 0.45 | 0.45 | 180 | 36 | 160 | 50.9 | 3.19 |
| Zhao et al [1] | 46.8 | 8.0 | 28 | 31.5 | 13 | 2.77 | 0.45 | 0.45 | 180 | 36 | 45 | 51.5 | 3.37 |
| Zhao et al [1] | 46.8 | 8.0 | 35 | 31.5 | 5 | 3.34 | 0.45 | 0.45 | 180 | 36 | 110 | 51.6 | 3.49 |
| Zhao et al [1] | 46.8 | 8.0 | 35 | 31.5 | 9 | 3.27 | 0.45 | 0.45 | 180 | 36 | 160 | 52.3 | 3.34 |
| Zhao et al [1] | 46.8 | 8.0 | 35 | 31.5 | 13 | 2.77 | 0.45 | 0.45 | 180 | 36 | 45 | 52.6 | 3.43 |
| Zhao et al [1] | 46.8 | 8.0 | 42 | 31.5 | 5 | 3.34 | 0.45 | 0.45 | 180 | 36 | 110 | 53.7 | 3.54 |
| Zhao et al [1] | 46.8 | 8.0 | 42 | 31.5 | 9 | 3.27 | 0.45 | 0.45 | 180 | 36 | 160 | 53.6 | 3.67 |
| Zhao et al [1] | 46.8 | 8.0 | 42 | 31.5 | 13 | 2.77 | 0.45 | 0.45 | 180 | 36 | 45 | 54.0 | 3.56 |
| Zhao et al [1] | 46.8 | 8.0 | 56 | 31.5 | 5 | 3.34 | 0.45 | 0.45 | 180 | 36 | 110 | 54.2 | 3.96 |
| Zhao et al [1] | 46.8 | 8.0 | 56 | 31.5 | 9 | 3.27 | 0.45 | 0.45 | 180 | 36 | 160 | 54.3 | 3.81 |
| Zhao et al [1] | 46.8 | 8.0 | 56 | 31.5 | 13 | 2.77 | 0.45 | 0.45 | 180 | 36 | 45 | 56.0 | 3.76 |
| Zhao et al [1] | 46.8 | 8.0 | 70 | 31.5 | 5 | 3.34 | 0.45 | 0.45 | 180 | 36 | 110 | 55.3 | 4.12 |
| Zhao et al [1] | 46.8 | 8.0 | 70 | 31.5 | 9 | 3.27 | 0.45 | 0.45 | 180 | 36 | 160 | 55.0 | 3.86 |
| Zhao et al [1] | 46.8 | 8.0 | 70 | 31.5 | 13 | 2.77 | 0.45 | 0.45 | 180 | 36 | 45 | 56.5 | 3.78 |
| Zhao et al [1] | 46.8 | 8.0 | 84 | 31.5 | 5 | 3.34 | 0.45 | 0.45 | 180 | 36 | 110 | 56.5 | 4.32 |
| Zhao et al [1] | 46.8 | 8.0 | 84 | 31.5 | 9 | 3.27 | 0.45 | 0.45 | 180 | 36 | 160 | 58.3 | 4.27 |
| Zhao et al [1] | 46.8 | 8.0 | 84 | 31.5 | 13 | 2.77 | 0.45 | 0.45 | 180 | 36 | 45 | 57.0 | 4.48 |
| Zhao et al [1] | 46.8 | 8.0 | 98 | 31.5 | 5 | 3.34 | 0.45 | 0.45 | 180 | 36 | 110 | 58.0 | 4.47 |
| Zhao et al [1] | 46.8 | 8.0 | 98 | 31.5 | 9 | 3.27 | 0.45 | 0.45 | 180 | 36 | 160 | 58.8 | 4.31 |
| Zhao et al [1] | 46.8 | 8.0 | 98 | 31.5 | 13 | 2.77 | 0.45 | 0.45 | 180 | 36 | 45 | 57.2 | 4.52 |
| Zhao et al [1] | 46.8 | 8.0 | 118 | 31.5 | 5 | 3.34 | 0.45 | 0.45 | 180 | 36 | 110 | 59.7 | 4.61 |
| Zhao et al [1] | 46.8 | 8.0 | 118 | 31.5 | 9 | 3.27 | 0.45 | 0.45 | 180 | 36 | 160 | 63.7 | 4.38 |
| Zhao et al [1] | 46.8 | 8.0 | 118 | 31.5 | 13 | 2.77 | 0.45 | 0.45 | 180 | 36 | 45 | 63.0 | 4.48 |
| Zhao et al [1] | 46.8 | 8.0 | 148 | 31.5 | 5 | 3.34 | 0.45 | 0.45 | 180 | 36 | 110 | 62.4 | 4.47 |
| Zhao et al [1] | 46.8 | 8.0 | 148 | 31.5 | 9 | 3.27 | 0.45 | 0.45 | 180 | 36 | 160 | 64.1 | 4.27 |
| Zhao et al [1] | 46.8 | 8.0 | 148 | 31.5 | 13 | 2.77 | 0.45 | 0.45 | 180 | 36 | 45 | 65.0 | 4.69 |
| Zhao et al [1] | 46.8 | 8.0 | 178 | 31.5 | 5 | 3.34 | 0.45 | 0.45 | 180 | 36 | 110 | 63.1 | 4.45 |
| Zhao et al [1] | 46.8 | 8.0 | 178 | 31.5 | 9 | 3.27 | 0.45 | 0.45 | 180 | 36 | 160 | 64.3 | 4.07 |
| Zhao et al [1] | 46.8 | 8.0 | 178 | 31.5 | 13 | 2.77 | 0.45 | 0.45 | 180 | 36 | 45 | 66.2 | 4.29 |
| Zhao et al [1] | 46.8 | 8.0 | 208 | 31.5 | 5 | 3.34 | 0.45 | 0.45 | 180 | 36 | 110 | 63.6 | 4.32 |
| Zhao et al [1] | 46.8 | 8.0 | 208 | 31.5 | 9 | 3.27 | 0.45 | 0.45 | 180 | 36 | 160 | 65.1 | 4.41 |
| Zhao et al [1] | 46.8 | 8.0 | 208 | 31.5 | 13 | 2.77 | 0.45 | 0.45 | 180 | 36 | 45 | 67.0 | 4.31 |
| Zhao et al [1] | 46.8 | 8.0 | 238 | 31.5 | 5 | 3.34 | 0.45 | 0.45 | 180 | 36 | 110 | 64.4 | 4.26 |
| Zhao et al [1] | 46.8 | 8.0 | 238 | 31.5 | 9 | 3.27 | 0.45 | 0.45 | 180 | 36 | 160 | 67.8 | 4.09 |
| Zhao et al [1] | 46.8 | 8.0 | 238 | 31.5 | 13 | 2.77 | 0.45 | 0.45 | 180 | 36 | 45 | 68.7 | 4.24 |
| Zhao et al [1] | 46.8 | 8.0 | 268 | 31.5 | 5 | 3.34 | 0.45 | 0.45 | 180 | 36 | 110 | 65.4 | 4.19 |
| Zhao et al [1] | 46.8 | 8.0 | 268 | 31.5 | 9 | 3.27 | 0.45 | 0.45 | 180 | 36 | 160 | 68.3 | 4.29 |
| Zhao et al [1] | 46.8 | 8.0 | 268 | 31.5 | 13 | 2.77 | 0.45 | 0.45 | 180 | 36 | 45 | 69.2 | 4.25 |
| Zhao et al [1] | 46.8 | 8.0 | 298 | 31.5 | 5 | 3.34 | 0.45 | 0.45 | 180 | 36 | 110 | 67.1 | 4.21 |
| Zhao et al [1] | 46.8 | 8.0 | 298 | 31.5 | 9 | 3.27 | 0.45 | 0.45 | 180 | 36 | 160 | 70.8 | 4.04 |
| Zhao et al [1] | 46.8 | 8.0 | 298 | 31.5 | 13 | 2.77 | 0.45 | 0.45 | 180 | 36 | 45 | 71.2 | 4.35 |
| Zhao et al [1] | 46.8 | 8.0 | 328 | 31.5 | 5 | 3.34 | 0.45 | 0.45 | 180 | 36 | 110 | 67.6 | 4.43 |
| Zhao et al [1] | 46.8 | 8.0 | 328 | 31.5 | 9 | 3.27 | 0.45 | 0.45 | 180 | 36 | 160 | 71.1 | 4.11 |
| Zhao et al [1] | 46.8 | 8.0 | 328 | 31.5 | 13 | 2.77 | 0.45 | 0.45 | 180 | 36 | 45 | 71.4 | 4.30 |
| Zhao et al [1] | 46.8 | 8.0 | 358 | 31.5 | 5 | 3.34 | 0.45 | 0.45 | 180 | 36 | 110 | 67.8 | 4.45 |
| Zhao et al [1] | 46.8 | 8.0 | 358 | 31.5 | 9 | 3.27 | 0.45 | 0.45 | 180 | 36 | 160 | 71.5 | 4.22 |
| Zhao et al [1] | 46.8 | 8.0 | 358 | 31.5 | 13 | 2.77 | 0.45 | 0.45 | 180 | 36 | 45 | 72.0 | 4.35 |
| Zhao et al [1] | 46.8 | 8.0 | 388 | 31.5 | 5 | 3.34 | 0.45 | 0.45 | 180 | 36 | 110 | 68.2 | 4.52 |
| Zhao et al [1] | 46.8 | 8.0 | 388 | 31.5 | 9 | 3.27 | 0.45 | 0.45 | 180 | 36 | 160 | 71.9 | 4.31 |
| Zhao et al [1] | 46.8 | 8.0 | 388 | 31.5 | 13 | 2.77 | 0.45 | 0.45 | 180 | 36 | 45 | 72.4 | 4.41 |
| Zhao et al [1] | 46.8 | 8.0 | 3 | 31.5 | 5 | 3.34 | 0.40 | 0.40 | 185 | 34 | 30 | 42.7 | 2.96 |
| Zhao et al [1] | 46.8 | 8.0 | 3 | 31.5 | 9 | 3.27 | 0.40 | 0.40 | 185 | 34 | 60 | 39.7 | 2.64 |
| Zhao et al [1] | 46.8 | 8.0 | 3 | 31.5 | 13 | 2.77 | 0.40 | 0.40 | 185 | 34 | 50 | 34.0 | 2.39 |
| Zhao et al [1] | 46.8 | 8.0 | 7 | 31.5 | 5 | 3.34 | 0.40 | 0.40 | 185 | 34 | 30 | 49.4 | 3.08 |
| Zhao et al [1] | 46.8 | 8.0 | 7 | 31.5 | 9 | 3.27 | 0.40 | 0.40 | 185 | 34 | 60 | 49.7 | 3.39 |
| Zhao et al [1] | 46.8 | 8.0 | 7 | 31.5 | 13 | 2.77 | 0.40 | 0.40 | 185 | 34 | 50 | 43.6 | 3.11 |
| Zhao et al [1] | 46.8 | 8.0 | 14 | 31.5 | 5 | 3.34 | 0.40 | 0.40 | 185 | 34 | 30 | 51.9 | 3.41 |
| Zhao et al [1] | 46.8 | 8.0 | 14 | 31.5 | 9 | 3.27 | 0.40 | 0.40 | 185 | 34 | 60 | 51.4 | 3.45 |
| Zhao et al [1] | 46.8 | 8.0 | 14 | 31.5 | 13 | 2.77 | 0.40 | 0.40 | 185 | 34 | 50 | 43.8 | 3.45 |
| Zhao et al [1] | 46.8 | 8.0 | 28 | 31.5 | 5 | 3.34 | 0.40 | 0.40 | 185 | 34 | 30 | 52.9 | 3.67 |
| Zhao et al [1] | 46.8 | 8.0 | 28 | 31.5 | 9 | 3.27 | 0.40 | 0.40 | 185 | 34 | 60 | 61.7 | 3.52 |
| Zhao et al [1] | 46.8 | 8.0 | 28 | 31.5 | 13 | 2.77 | 0.40 | 0.40 | 185 | 34 | 50 | 57.7 | 3.75 |
| Zhao et al [1] | 46.8 | 8.0 | 42 | 31.5 | 5 | 3.34 | 0.40 | 0.40 | 185 | 34 | 30 | 64.0 | 3.94 |
| Zhao et al [1] | 46.8 | 8.0 | 42 | 31.5 | 9 | 3.27 | 0.40 | 0.40 | 185 | 34 | 60 | 62.5 | 3.81 |
| Zhao et al [1] | 46.8 | 8.0 | 42 | 31.5 | 13 | 2.77 | 0.40 | 0.40 | 185 | 34 | 50 | 60.6 | 3.79 |
| Zhao et al [1] | 46.8 | 8.0 | 56 | 31.5 | 5 | 3.34 | 0.40 | 0.40 | 185 | 34 | 30 | 65.0 | 3.97 |
| Zhao et al [1] | 46.8 | 8.0 | 56 | 31.5 | 9 | 3.27 | 0.40 | 0.40 | 185 | 34 | 60 | 64.0 | 4.03 |
| Zhao et al [1] | 46.8 | 8.0 | 56 | 31.5 | 13 | 2.77 | 0.40 | 0.40 | 185 | 34 | 50 | 61.2 | 3.99 |
| Zhao et al [1] | 46.8 | 8.0 | 84 | 31.5 | 5 | 3.34 | 0.40 | 0.40 | 185 | 34 | 30 | 66.9 | 4.02 |
| Zhao et al [1] | 46.8 | 8.0 | 84 | 31.5 | 9 | 3.27 | 0.40 | 0.40 | 185 | 34 | 60 | 64.6 | 4.42 |
| Zhao et al [1] | 46.8 | 8.0 | 84 | 31.5 | 13 | 2.77 | 0.40 | 0.40 | 185 | 34 | 50 | 63.0 | 4.35 |
| Zhao et al [1] | 46.8 | 8.0 | 118 | 31.5 | 5 | 3.34 | 0.40 | 0.40 | 185 | 34 | 30 | 67.3 | 4.37 |
| Zhao et al [1] | 46.8 | 8.0 | 118 | 31.5 | 9 | 3.27 | 0.40 | 0.40 | 185 | 34 | 60 | 65.0 | 4.47 |
| Zhao et al [1] | 46.8 | 8.0 | 118 | 31.5 | 13 | 2.77 | 0.40 | 0.40 | 185 | 34 | 50 | 64.0 | 4.03 |
| Zhao et al [1] | 46.8 | 8.0 | 178 | 31.5 | 5 | 3.34 | 0.40 | 0.40 | 185 | 34 | 30 | 68.0 | 4.68 |
| Zhao et al [1] | 46.8 | 8.0 | 178 | 31.5 | 9 | 3.27 | 0.40 | 0.40 | 185 | 34 | 60 | 69.6 | 4.33 |
| Zhao et al [1] | 46.8 | 8.0 | 178 | 31.5 | 13 | 2.77 | 0.40 | 0.40 | 185 | 34 | 50 | 67.8 | 3.99 |
| Zhao et al [1] | 46.8 | 8.0 | 238 | 31.5 | 5 | 3.34 | 0.40 | 0.40 | 185 | 34 | 30 | 73.0 | 4.55 |
| Zhao et al [1] | 46.8 | 8.0 | 238 | 31.5 | 9 | 3.27 | 0.40 | 0.40 | 185 | 34 | 60 | 71.0 | 4.02 |
| Zhao et al [1] | 46.8 | 8.0 | 238 | 31.5 | 13 | 2.77 | 0.40 | 0.40 | 185 | 34 | 50 | 68.0 | 4.05 |
| Zhao et al [1] | 46.8 | 8.0 | 298 | 31.5 | 5 | 3.34 | 0.40 | 0.40 | 185 | 34 | 30 | 74.1 | 4.59 |
| Zhao et al [1] | 46.8 | 8.0 | 298 | 31.5 | 9 | 3.27 | 0.40 | 0.40 | 185 | 34 | 60 | 77.9 | 4.11 |
| Zhao et al [1] | 46.8 | 8.0 | 298 | 31.5 | 13 | 2.77 | 0.40 | 0.40 | 185 | 34 | 50 | 70.6 | 4.18 |
| Zhao et al [1] | 46.8 | 8.0 | 358 | 31.5 | 5 | 3.34 | 0.40 | 0.40 | 185 | 34 | 30 | 74.6 | 4.51 |
| Zhao et al [1] | 46.8 | 8.0 | 358 | 31.5 | 9 | 3.27 | 0.40 | 0.40 | 185 | 34 | 60 | 78.2 | 4.24 |
| Zhao et al [1] | 46.8 | 8.0 | 358 | 31.5 | 13 | 2.77 | 0.40 | 0.40 | 185 | 34 | 50 | 73.1 | 4.34 |
| Li et al. [2] | 38.2 | 6.9 | 28 | 25 | 3 | 3.19 | 0.47 | 0.47 | 190 | 36 | 22 | 47.1 | 2.95 |
| Li et al. [2] | 38.2 | 6.9 | 28 | 25 | 5 | 3.19 | 0.47 | 0.47 | 190 | 36 | 16 | 40.7 | 3.16 |
| Li et al. [2] | 38.2 | 6.9 | 28 | 25 | 7 | 3.19 | 0.47 | 0.47 | 190 | 36 | 28 | 44.3 | 3.44 |
| Li et al. [2] | 38.2 | 6.9 | 28 | 25 | 10 | 3.19 | 0.47 | 0.47 | 190 | 36 | 11 | 45.9 | 3.57 |
| Li et al. [2] | 38.2 | 6.9 | 28 | 25 | 13 | 3.19 | 0.47 | 0.47 | 190 | 36 | 17 | 43.4 | 3.60 |
| Li et al. [2] | 38.2 | 6.9 | 28 | 25 | 16 | 3.19 | 0.47 | 0.47 | 190 | 36 | 12 | 44.3 | 3.58 |
| Li et al. [2] | 47.7 | 8.7 | 28 | 25 | 3 | 3.19 | 0.32 | 0.32 | 170 | 30 | 20 | 69.6 | 4.00 |
| Li et al. [2] | 47.7 | 8.7 | 28 | 25 | 5 | 3.19 | 0.32 | 0.32 | 170 | 30 | 11 | 71.6 | 4.10 |
| Li et al. [2] | 47.7 | 8.7 | 28 | 25 | 7 | 3.19 | 0.32 | 0.32 | 170 | 30 | 13 | 74.7 | 4.34 |
| Li et al. [2] | 47.7 | 8.7 | 28 | 25 | 10 | 3.19 | 0.32 | 0.32 | 170 | 30 | 35 | 69.8 | 3.90 |
| Li et al. [2] | 47.7 | 8.7 | 28 | 25 | 13 | 3.19 | 0.32 | 0.32 | 170 | 30 | 20 | 69.8 | 4.37 |
| Li et al. [2] | 47.7 | 8.7 | 28 | 25 | 16 | 3.19 | 0.32 | 0.32 | 170 | 30 | 18 | 70.1 | 4.20 |
| Li et al. [2] | 47.7 | 8.7 | 28 | 25 | 5 | 3.19 | 0.44 | 0.44 | 180 | 32 | 85 | 57 | 3.68 |
| Li et al. [2] | 47.7 | 8.7 | 28 | 25 | 7 | 3.19 | 0.44 | 0.44 | 180 | 32 | 108 | 59.6 | 3.91 |
| Li et al. [2] | 47.7 | 8.7 | 28 | 25 | 10 | 3.19 | 0.44 | 0.44 | 180 | 32 | 75 | 56.7 | 4.11 |
| Li et al. [2] | 47.7 | 8.7 | 28 | 25 | 13 | 3.19 | 0.44 | 0.44 | 180 | 32 | 80 | 57.0 | 3.42 |
| Li et al. [2] | 47.7 | 8.7 | 28 | 25 | 16 | 3.19 | 0.44 | 0.44 | 180 | 32 | 60 | 56.2 | 2.86 |
| Li et al. [2] | 46.3 | 8.4 | 28 | 25 | 5 | 3.19 | 0.56 | 0.56 | 170 | 42 | 135 | 42.9 | 3.34 |
| Li et al. [2] | 46.3 | 8.4 | 28 | 25 | 9 | 3.19 | 0.56 | 0.56 | 170 | 42 | 80 | 43.8 | 3.31 |
| Li et al. [2] | 46.3 | 8.4 | 28 | 25 | 13 | 3.19 | 0.56 | 0.56 | 170 | 42 | 50 | 43.9 | 2.76 |
| Li et al. [2] | 46.3 | 8.4 | 28 | 25 | 5 | 3.19 | 0.45 | 0.45 | 185 | 34 | 70 | 48.7 | 3.11 |
| Li et al. [2] | 46.3 | 8.4 | 28 | 25 | 9 | 3.19 | 0.45 | 0.45 | 185 | 34 | 160 | 50.1 | 3.51 |
| Li et al. [2] | 46.3 | 8.4 | 28 | 25 | 13 | 3.19 | 0.45 | 0.45 | 185 | 34 | 100 | 56.3 | 4.61 |
| Li et al. [2] | 46.3 | 8.4 | 28 | 25 | 5 | 3.19 | 0.4 | 0.4 | 190 | 32 | 150 | 55.6 | 4.19 |
| Li et al. [2] | 46.3 | 8.4 | 28 | 25 | 9 | 3.19 | 0.4 | 0.4 | 190 | 32 | 170 | 60.7 | 4.33 |
| Li et al. [2] | 46.3 | 8.4 | 28 | 25 | 13 | 3.19 | 0.4 | 0.4 | 190 | 32 | 175 | 57.2 | 2.45 |
| Li et al. [2] | 46.3 | 8.4 | 28 | 25 | 5 | 3.19 | 0.32 | 0.32 | 175 | 28 | 50 | 70.9 | 3.95 |
| Li et al. [2] | 46.3 | 8.4 | 28 | 25 | 9 | 3.19 | 0.32 | 0.32 | 175 | 28 | 110 | 68.1 | 4.23 |
| Li et al. [2] | 46.3 | 8.4 | 28 | 25 | 13 | 3.19 | 0.32 | 0.32 | 175 | 28 | 100 | 66.7 | 5.45 |
| Li et al. [2] | 38.2 | 6.9 | 28 | 26.5 | 3 | 3.23 | 0.47 | 0.47 | 190 | 36 | 12 | 40.3 | 3.32 |
| Li et al. [2] | 38.2 | 6.9 | 28 | 26.5 | 7 | 3.23 | 0.47 | 0.47 | 190 | 36 | 19 | 38.7 | 3.14 |
| Li et al. [2] | 38.2 | 6.9 | 28 | 26.5 | 13 | 3.23 | 0.47 | 0.47 | 190 | 36 | 15 | 40.7 | 2.96 |
| Li et al. [2] | 47.7 | 8.7 | 28 | 26.5 | 3 | 3.23 | 0.44 | 0.44 | 180 | 32 | 75 | 50.4 | 3.65 |
| Li et al. [2] | 47.7 | 8.7 | 28 | 26.5 | 7 | 3.23 | 0.44 | 0.44 | 180 | 32 | 83 | 50.3 | 3.88 |
| Li et al. [2] | 47.7 | 8.7 | 28 | 26.5 | 13 | 3.23 | 0.44 | 0.44 | 180 | 32 | 60 | 55.4 | 4.08 |
| Li et al. [2] | 47.7 | 8.7 | 28 | 26.5 | 3 | 3.23 | 0.32 | 0.32 | 170 | 30 | 11 | 62.6 | 4.57 |
| Li et al. [2] | 47.7 | 8.7 | 28 | 26.5 | 7 | 3.23 | 0.32 | 0.32 | 170 | 30 | 15 | 63.1 | 5.16 |
| Li et al. [2] | 47.7 | 8.7 | 28 | 26.5 | 13 | 3.23 | 0.32 | 0.32 | 170 | 30 | 18 | 59.9 | 5.32 |
| Li et al. [2] | 48.2 | 8.4 | 28 | 26.5 | 5 | 3.23 | 0.55 | 0.55 | 160 | 38 | 115 | 43.4 | 2.96 |
| Li et al. [2] | 48.2 | 8.4 | 28 | 26.5 | 9 | 3.23 | 0.55 | 0.55 | 160 | 38 | 125 | 44.2 | 3.45 |
| Li et al. [2] | 48.2 | 8.4 | 28 | 26.5 | 13 | 3.23 | 0.55 | 0.55 | 160 | 38 | 22 | 43.2 | 3.20 |
| Li et al. [2] | 48.2 | 8.4 | 28 | 26.5 | 5 | 3.23 | 0.44 | 0.44 | 170 | 34 | 30 | 55 | 3.30 |
| Li et al. [2] | 48.2 | 8.4 | 28 | 26.5 | 9 | 3.23 | 0.44 | 0.44 | 170 | 34 | 135 | 52.4 | 3.47 |
| Li et al. [2] | 48.2 | 8.4 | 28 | 26.5 | 13 | 3.23 | 0.44 | 0.44 | 170 | 34 | 90 | 53.5 | 3.18 |
| Li et al. [2] | 48.2 | 8.4 | 28 | 26.5 | 5 | 3.23 | 0.36 | 0.36 | 170 | 32 | 95 | 59.9 | 3.51 |
| Li et al. [2] | 48.2 | 8.4 | 28 | 26.5 | 9 | 3.23 | 0.36 | 0.36 | 170 | 32 | 85 | 57.2 | 5.24 |
| Li et al. [2] | 48.2 | 8.4 | 28 | 26.5 | 13 | 3.23 | 0.36 | 0.36 | 170 | 32 | 70 | 59.6 | 4.53 |
| Li et al. [2] | 48.2 | 8.4 | 28 | 26.5 | 5 | 3.23 | 0.31 | 0.31 | 170 | 30 | 35 | 68.2 | 3.62 |
| Li et al. [2] | 48.2 | 8.4 | 28 | 26.5 | 9 | 3.23 | 0.31 | 0.31 | 170 | 30 | 20 | 68.3 | 3.99 |
| Li et al. [2] | 48.2 | 8.4 | 28 | 26.5 | 13 | 3.23 | 0.31 | 0.31 | 170 | 30 | 25 | 66 | 4.36 |
| Li et al. [2] | 46.3 | 8.4 | 28 | 26.5 | 6.75 | 3.19 | 0.56 | 0.56 | 170 | 42 | - | 39.2 | 3.47 |
| Li et al. [2] | 46.3 | 8.4 | 28 | 26.5 | 6.75 | 3.19 | 0.44 | 0.44 | 180 | 32 | - | 50.1 | 3.77 |
| Li et al. [2] | 46.3 | 8.4 | 28 | 26.5 | 6.75 | 3.19 | 0.32 | 0.32 | 170 | 28 | - | 59.1 | 4.31 |
| Chai et al. [3] | 48.3 | 7.4 | 28 | 25 | 7.5 | 2.9 | 0.51 | 1.01 | 222 | 45 | - | 57.0 | 2.87 |
| He et al. [4, 5] | - | - | 28 | 30 | 4.3 | 3.0 | 0.25 | 0.33 | 150 | 41 | - | 74.4 | 5.41 |
| He et al. [4,5] | - | - | 60 | 30 | 4.3 | 3.0 | 0.25 | 0.33 | 150 | 41 | - | 84.5 | 6.37 |
| He et al. [4,5] | - | - | 90 | 30 | 4.3 | 3.0 | 0.25 | 0.33 | 150 | 41 | - | 94.1 | 6.50 |
| He et al. [4,5] | - | - | 120 | 30 | 4.3 | 3.0 | 0.25 | 0.33 | 150 | 41 | - | 100.5 | 6.65 |
| Jiang [6] | - | - | 28 | 31.5 | 5.8 | 3.1 | 0.42 | 0.42 | 185 | 36 | 36 | 38.3 | 3.04 |
| Jiang [6] | - | - | 28 | 31.5 | 5.8 | 3.1 | 0.44 | 0.44 | 185 | 36 | 40 | 36.2 | 2.75 |
| Jiang [6] | - | - | 28 | 31.5 | 5.8 | 3.1 | 0.46 | 0.46 | 185 | 36 | 43 | 34.2 | 2.60 |
| Jiang [6] | - | - | 28 | 31.5 | 5.8 | 3.1 | 0.42 | 0.42 | 185 | 32 | 25 | 35.9 | 2.42 |
| Jiang [6] | - | - | 28 | 31.5 | 5.8 | 3.1 | 0.42 | 0.42 | 185 | 40 | 40 | 37.3 | 3.24 |
| Li et al [7] | - | - | 28 | - | - | - | 0.45 | 0.45 | 180 | 39 | 205 | 47.4 | 4.98 |
| Li et al [7] | - | - | 28 | - | - | - | 0.5 | 0.5 | 180 | 39 | 205 | 45.3 | 4.68 |
| Li et al [7] | - | - | 28 | - | - | - | 0.55 | 0.55 | 180 | 40 | 200 | 41.7 | 4.5 |
| Li and Zhang [8] | - | - | 28 | - | 4.3 | 2.86 | - | - | - | - | - | 35.8 | 1.63 |
| Li and Zhang [8] | - | - | 28 | - | 7.5 | 3.04 | - | - | - | - | - | 36.5 | 1.77 |
| Li and Zhang [8] | - | - | 28 | - | 10.8 | 2.76 | - | - | - | - | - | 37.8 | 1.84 |
| Li and Zhang [8] | - | - | 28 | - | 19.2 | 2.35 | - | - | - | - | - | 34.3 | 1.72 |
| Shi [9] | 35.5 | 7.2 | 28 | 30 | 14.2 | 2.2 | 0.57 | 0.57 | 178 | 35 | 20 | 27.9 | 1.97 |
| Shi [9] | 35.5 | 7.2 | 28 | 30 | 14.2 | 2.2 | 0.64 | 0.64 | 178 | 35 | 20 | 24.7 | 2.05 |
| Shi [9] | 35.5 | 7.2 | 28 | 30 | 14.2 | 2.2 | 0.69 | 0.69 | 178 | 35 | 20 | 22.8 | 2.11 |
| Wang and Xu [10] | - | - | 28 | 20 | 13 | 3.55 | 0.59 | 0.59 | 203 | 40 | 70 | 43.1 | 2.65 |
| Wang and Xu [10] | - | - | 28 | 20 | 7.8 | 3.55 | 0.63 | 0.63 | 241 | 40 | 140 | 38.9 | 2.18 |
| Wang and Xu [10] | - | - | 28 | 20 | 16.3 | 3.55 | 0.56 | 0.56 | 185 | 39 | 100 | 39.0 | 2.87 |
| Wang and Xu [10] | - | - | 28 | 20 | 11.3 | 3.55 | 0.47 | 0.47 | 196 | 43 | 60 | 49.0 | 2.19 |
| Yang [11] | 63.4 | 10.2 | 28 | 16 | 3 | 3.5 | 0.25 | 0.48 | 150 | 37 | 240 | 94.3 | 6.2 |
| Yang [11] | 63.4 | 10.2 | 28 | 16 | 5 | 3.5 | 0.25 | 0.48 | 150 | 37 | 225 | 94.6 | 6.4 |
| Yang [11] | 63.4 | 10.2 | 28 | 16 | 7 | 3.5 | 0.25 | 0.48 | 150 | 37 | 220 | 96.3 | 5.4 |
| Yang [11] | 63.4 | 10.2 | 28 | 16 | 10 | 3.5 | 0.25 | 0.48 | 150 | 37 | 180 | 91.0 | 5.6 |
| Zhang [12] | 55.6 | 8.91 | 1 | 20 | 19.4 | 2.80 | 0.28 | 0.40 | 180 | 45 | - | 4.23 | 0.6 |
| Zhang [12] | 55.6 | 8.91 | 3 | 20 | 19.4 | 2.80 | 0.28 | 0.40 | 180 | 45 | - | 14.9 | 1.35 |
| Zhang [12] | 55.6 | 8.91 | 7 | 20 | 19.4 | 2.80 | 0.28 | 0.40 | 180 | 45 | - | 28.3 | 2.24 |
| Zhang [12] | 55.6 | 8.91 | 14 | 20 | 19.4 | 2.80 | 0.28 | 0.40 | 180 | 45 | - | 40.9 | 3.01 |
| Zhang [12] | 55.6 | 8.91 | 28 | 20 | 19.4 | 2.80 | 0.28 | 0.40 | 180 | 45 | - | 50.8 | 3.64 |
| Zhang [12] | 55.6 | 8.91 | 60 | 20 | 19.4 | 2.80 | 0.28 | 0.40 | 180 | 45 | - | 59.5 | 4.32 |
| Zhang [12] | 55.6 | 8.91 | 90 | 20 | 19.4 | 2.80 | 0.28 | 0.40 | 180 | 45 | - | 65.4 | 4.43 |
| Lu [13] | - | - | 28 | 40 | 7.1* | - | 0.45 | 0.56 | 109 | 36 | 85 | 24.8 | 1.98 |
| Lu [13] | - | - | 28 | 40 | 9.1* | - | 0.45 | 0.56 | 111 | 36 | 86 | 28.1 | 2.23 |
| Lu [13] | - | - | 28 | 40 | 11.2* | - | 0.45 | 0.56 | 113 | 36 | 83 | 29.8 | 2.41 |
| Lu [13] | - | - | 28 | 40 | 13.3* | - | 0.45 | 0.56 | 116 | 36 | 85 | 31.5 | 2.47 |
| Lu [13] | - | - | 28 | 40 | 15.5* | - | 0.45 | 0.56 | 119 | 36 | 90 | 34 | 2.65 |
| Lu [13] | - | - | 28 | 40 | 17.8* | - | 0.45 | 0.56 | 122 | 36 | 88 | 33.2 | 2.68 |
| Lu [13] | - | - | 28 | 40 | 20.0* | - | 0.45 | 0.56 | 124 | 36 | 87 | 32.8 | 2.54 |
| Lu [13] | - | - | 28 | 40 | 22.1* | - | 0.45 | 0.56 | 126 | 36 | 85 | 31.6 | 2.37 |
| Lu [13] | - | - | 28 | 40 | 7.1* | - | 0.45 | 0.56 | 129 | 42 | 171 | 24.1 | 2.01 |
| Lu [13] | - | - | 28 | 40 | 9.1* | - | 0.45 | 0.56 | 131 | 42 | 177 | 27.5 | 2.36 |
| Lu [13] | - | - | 28 | 40 | 11.2* | - | 0.45 | 0.56 | 133 | 42 | 182 | 29 | 2.37 |
| Lu [13] | - | - | 28 | 40 | 13.3* | - | 0.45 | 0.56 | 136 | 42 | 176 | 30.9 | 2.41 |
| Lu [13] | - | - | 28 | 40 | 15.5* | - | 0.45 | 0.56 | 139 | 42 | 175 | 33.7 | 2.47 |
| Lu [13] | - | - | 28 | 40 | 17.8* | - | 0.45 | 0.56 | 142 | 42 | 179 | 32.4 | 2.56 |
| Lu [13] | - | - | 28 | 40 | 20.0* | - | 0.45 | 0.56 | 144 | 42 | 180 | 31.2 | 2.52 |
| Lu [13] | - | - | 28 | 40 | 22.1* | - | 0.45 | 0.56 | 146 | 42 | 173 | 30.9 | 2.41 |
| Li et al. [14] | - | - | 7 | 40 | 6* | - | 0.36 | 0.48 | 126 | 35 | 50 | 24.6 | 1.95 |
| Li et al. [14] | - | - | 7 | 40 | 9* | - | 0.36 | 0.48 | 128 | 34 | 45 | 25.3 | 2.25 |
| Li et al. [14] | - | - | 7 | 40 | 12* | - | 0.36 | 0.48 | 130 | 34 | 60 | 25.0 | 2.09 |
| Li et al. [14] | - | - | 7 | 40 | 15* | - | 0.36 | 0.48 | 131 | 33 | 55 | 24.8 | 1.97 |
| Li et al. [14] | - | - | 7 | 40 | 18* | - | 0.36 | 0.48 | 135 | 33 | 60 | 26.1 | 2.29 |
| Li et al. [14] | - | - | 28 | 40 | 6* | - | 0.36 | 0.48 | 126 | 35 | 50 | 40.1 | 3.13 |
| Li et al. [14] | - | - | 28 | 40 | 9* | - | 0.36 | 0.48 | 128 | 34 | 45 | 41.8 | 2.88 |
| Li et al. [14] | - | - | 28 | 40 | 12* | - | 0.36 | 0.48 | 130 | 34 | 60 | 41.4 | 3.03 |
| Li et al. [14] | - | - | 28 | 40 | 15* | - | 0.36 | 0.48 | 131 | 33 | 55 | 41.3 | 3.21 |
| Li et al. [14] | - | - | 28 | 40 | 18* | - | 0.36 | 0.48 | 135 | 33 | 60 | 41.1 | 3.07 |
| Li et al. [14] | - | - | 90 | 40 | 6* | - | 0.36 | 0.48 | 126 | 35 | 50 | 55.1 | 3.82 |
| Li et al. [14] | - | - | 90 | 40 | 9* | - | 0.36 | 0.48 | 128 | 34 | 45 | 56.1 | 3.90 |
| Li et al. [14] | - | - | 90 | 40 | 12* | - | 0.36 | 0.48 | 130 | 34 | 60 | 51.0 | 3.79 |
| Li et al. [14] | - | - | 90 | 40 | 15* | - | 0.36 | 0.48 | 131 | 33 | 55 | 51.5 | 3.77 |
| Li et al. [14] | - | - | 90 | 40 | 18* | - | 0.36 | 0.48 | 135 | 33 | 60 | 53.2 | 4.12 |
| Li et al. [14] | - | - | 180 | 40 | 6* | - | 0.36 | 0.48 | 126 | 35 | 50 | 54.8 | 3.97 |
| Li et al. [14] | - | - | 180 | 40 | 9* | - | 0.36 | 0.48 | 128 | 34 | 45 | 61.6 | 4.45 |
| Li et al. [14] | - | - | 180 | 40 | 12* | - | 0.36 | 0.48 | 130 | 34 | 60 | 54.6 | 4.51 |
| Li et al. [14] | - | - | 180 | 40 | 15* | - | 0.36 | 0.48 | 131 | 33 | 55 | 54.8 | 4.02 |
| Li et al. [14] | - | - | 180 | 40 | 18* | - | 0.36 | 0.48 | 135 | 33 | 60 | 58.8 | 4.5 |
| Chen [15] | 52.5 | 8.8 | 3 | 20 | 5.4 | 3.11 | 0.37 | 0.44 | 166 | 44 | 185 | 19.0 | 1.6 |
| Chen [15] | 52.5 | 8.8 | 7 | 20 | 5.4 | 3.11 | 0.37 | 0.44 | 166 | 44 | 185 | 31.5 | 2.4 |
| Chen [15] | 52.5 | 8.8 | 28 | 20 | 5.4 | 3.11 | 0.37 | 0.44 | 166 | 44 | 185 | 45.8 | 2.6 |
| Chen [15] | 52.5 | 8.8 | 56 | 20 | 5.4 | 3.11 | 0.37 | 0.44 | 166 | 44 | 185 | 53.0 | 3.6 |
| Chen [15] | 52.5 | 8.8 | 3 | 20 | 5.4 | 3.11 | 0.34 | 0.34 | 158 | 42 | 105 | 48.2 | 3.6 |
| Chen [15] | 52.5 | 8.8 | 7 | 20 | 5.4 | 3.11 | 0.34 | 0.34 | 158 | 42 | 105 | 56.6 | 3.8 |
| Chen [15] | 52.5 | 8.8 | 28 | 20 | 5.4 | 3.11 | 0.34 | 0.34 | 158 | 42 | 105 | 69.1 | 4.0 |
| Chen [15] | 52.5 | 8.8 | 56 | 20 | 5.4 | 3.11 | 0.34 | 0.34 | 158 | 42 | 105 | 69.4 | 4.4 |
| Lin [16] | 52.7 | 9.0 | 3 | 31.5 | 6.6 | 2.94 | 0.42 | 0.42 | 185 | 36 | 35 | 23.4 | 2.03 |
| Lin [16] | 52.7 | 9.0 | 28 | 31.5 | 6.6 | 2.94 | 0.42 | 0.42 | 185 | 36 | 35 | 35.6 | 3.04 |
| Lin [16] | 52.7 | 9.0 | 3 | 31.5 | 6.6 | 2.94 | 0.44 | 0.44 | 185 | 36 | 41 | 23.7 | 1.93 |
| Lin [16] | 52.7 | 9.0 | 28 | 31.5 | 6.6 | 2.94 | 0.44 | 0.44 | 185 | 36 | 41 | 32.2 | 2.74 |
| Lin [16] | 52.7 | 9.0 | 3 | 31.5 | 6.6 | 2.94 | 0.46 | 0.46 | 185 | 36 | 42 | 22.5 | 2.09 |
| Lin [16] | 52.7 | 9.0 | 28 | 31.5 | 6.6 | 2.94 | 0.46 | 0.46 | 185 | 36 | 42 | 34.2 | 2.59 |
| Lin [16] | 52.7 | 9.0 | 3 | 31.5 | 6.6 | 2.94 | 0.42 | 0.42 | 185 | 32 | 25 | 23.6 | 1.69 |
| Lin [16] | 52.7 | 9.0 | 28 | 31.5 | 6.6 | 2.94 | 0.42 | 0.42 | 185 | 32 | 25 | 35.8 | 2.43 |
| Lin [16] | 52.7 | 9.0 | 3 | 31.5 | 6.6 | 2.94 | 0.44 | 0.44 | 185 | 34 | 33 | 25.2 | 1.52 |
| Lin [16] | 52.7 | 9.0 | 28 | 31.5 | 6.6 | 2.94 | 0.44 | 0.44 | 185 | 34 | 33 | 38.3 | 2.6 |
| Lin [16] | 52.7 | 9.0 | 3 | 31.5 | 6.6 | 2.94 | 0.46 | 0.46 | 185 | 36 | 40 | 23.3 | 1.80 |
| Lin [16] | 52.7 | 9.0 | 28 | 31.5 | 6.6 | 2.94 | 0.46 | 0.46 | 185 | 36 | 40 | 37.2 | 3.24 |
| Lin [17] | 43.7 | 7.5 | 3 | 25 | 0.4 | 2.3 | 0.3 | 0.425 | 153 | 41 | 220 | 46.3 | 3.04 |
| Lin [17] | 43.7 | 7.5 | 7 | 25 | 0.4 | 2.3 | 0.3 | 0.425 | 153 | 41 | 220 | 58.5 | 3.53 |
| Lin [17] | 43.7 | 7.5 | 28 | 25 | 0.4 | 2.3 | 0.3 | 0.425 | 153 | 41 | 220 | 72.7 | 4.15 |
| Lin [17] | 43.7 | 7.5 | 60 | 25 | 0.4 | 2.3 | 0.3 | 0.425 | 153 | 41 | 220 | 76.8 | 4.59 |
| Lin [17] | 43.7 | 7.5 | 90 | 25 | 0.4 | 2.3 | 0.3 | 0.425 | 153 | 41 | 220 | 80.8 | 4.82 |
| Lin [17] | 43.7 | 7.5 | 120 | 25 | 0.4 | 2.3 | 0.3 | 0.425 | 153 | 41 | 220 | 83.8 | 5.03 |
| Lin [17] | 43.7 | 7.5 | 180 | 25 | 0.4 | 2.3 | 0.3 | 0.425 | 153 | 41 | 220 | 84.6 | 5.07 |
| Lin [17] | 43.7 | 7.5 | 365 | 25 | 0.4 | 2.3 | 0.3 | 0.425 | 153 | 41 | 220 | 87.2 | 5.14 |
| Lin [17] | 43.7 | 7.5 | 3 | 25 | 2.3 | 3.1 | 0.3 | 0.428 | 152 | 38 | 210 | 46.2 | 3.19 |
| Lin [17] | 43.7 | 7.5 | 7 | 25 | 2.3 | 3.1 | 0.3 | 0.428 | 152 | 38 | 210 | 56.9 | 3.58 |
| Lin [17] | 43.7 | 7.5 | 28 | 25 | 2.3 | 3.1 | 0.3 | 0.428 | 152 | 38 | 210 | 74.5 | 4.36 |
| Lin [17] | 43.7 | 7.5 | 60 | 25 | 2.3 | 3.1 | 0.3 | 0.428 | 152 | 38 | 210 | 79.3 | 4.65 |
| Lin [17] | 43.7 | 7.5 | 90 | 25 | 2.3 | 3.1 | 0.3 | 0.428 | 152 | 38 | 210 | 81.3 | 4.89 |
| Lin [17] | 43.7 | 7.5 | 120 | 25 | 2.3 | 3.1 | 0.3 | 0.428 | 152 | 38 | 210 | 82.6 | 4.99 |
| Lin [17] | 43.7 | 7.5 | 180 | 25 | 2.3 | 3.1 | 0.3 | 0.428 | 152 | 38 | 210 | 83.5 | 5.05 |
| Lin [17] | 43.7 | 7.5 | 365 | 25 | 2.3 | 3.1 | 0.3 | 0.428 | 152 | 38 | 210 | 85.2 | 5.17 |
| Lin [17] | 43.7 | 7.5 | 3 | 25 | - | 3.1 | 0.3 | 0.428 | 152 | 38 | 215 | 48.5 | 3.24 |
| Lin [17] | 43.7 | 7.5 | 7 | 25 | - | 3.1 | 0.3 | 0.428 | 152 | 38 | 215 | 60.4 | 3.8 |
| Lin [17] | 43.7 | 7.5 | 28 | 25 | - | 3.1 | 0.3 | 0.428 | 152 | 38 | 215 | 75.9 | 4.49 |
| Lin [17] | 43.7 | 7.5 | 60 | 25 | - | 3.1 | 0.3 | 0.428 | 152 | 38 | 215 | 82.1 | 4.78 |
| Lin [17] | 43.7 | 7.5 | 90 | 25 | - | 3.1 | 0.3 | 0.428 | 152 | 38 | 215 | 84.2 | 4.92 |
| Lin [17] | 43.7 | 7.5 | 120 | 25 | - | 3.1 | 0.3 | 0.428 | 152 | 38 | 215 | 86.1 | 5.05 |
| Lin [17] | 43.7 | 7.5 | 180 | 25 | - | 3.1 | 0.3 | 0.428 | 152 | 38 | 215 | 88.6 | 5.18 |
| Lin [17] | 43.7 | 7.5 | 365 | 25 | - | 3.1 | 0.3 | 0.428 | 152 | 38 | 215 | 90.7 | 5.39 |
| Lin [17] | 43.7 | 7.5 | 3 | 25 | 6.3 | 3.1 | 0.3 | 0.428 | 152 | 38 | 202 | 50.7 | 3.42 |
| Lin [17] | 43.7 | 7.5 | 7 | 25 | 6.3 | 3.1 | 0.3 | 0.428 | 152 | 38 | 202 | 61 | 3.9 |
| Lin [17] | 43.7 | 7.5 | 28 | 25 | 6.3 | 3.1 | 0.3 | 0.428 | 152 | 38 | 202 | 79.5 | 4.73 |
| Lin [17] | 43.7 | 7.5 | 60 | 25 | 6.3 | 3.1 | 0.3 | 0.428 | 152 | 38 | 202 | 85.4 | 5.05 |
| Lin [17] | 43.7 | 7.5 | 90 | 25 | 6.3 | 3.1 | 0.3 | 0.428 | 152 | 38 | 202 | 87.3 | 5.18 |
| Lin [17] | 43.7 | 7.5 | 120 | 25 | 6.3 | 3.1 | 0.3 | 0.428 | 152 | 38 | 202 | 88.5 | 5.24 |
| Lin [17] | 43.7 | 7.5 | 180 | 25 | 6.3 | 3.1 | 0.3 | 0.428 | 152 | 38 | 202 | 90.2 | 5.32 |
| Lin [17] | 43.7 | 7.5 | 365 | 25 | 6.3 | 3.1 | 0.3 | 0.428 | 152 | 38 | 202 | 92.2 | 5.48 |
| Lin [17] | 43.7 | 7.5 | 3 | 25 | - | 3.1 | 0.3 | 0.3 | 152 | 38 | 205 | 58.7 | 3.54 |
| Lin [17] | 43.7 | 7.5 | 7 | 25 | - | 3.1 | 0.3 | 0.3 | 152 | 38 | 205 | 66.7 | 3.98 |
| Lin [17] | 43.7 | 7.5 | 28 | 25 | - | 3.1 | 0.3 | 0.3 | 152 | 38 | 205 | 78.8 | 4.61 |
| Lin [17] | 43.7 | 7.5 | 60 | 25 | - | 3.1 | 0.3 | 0.3 | 152 | 38 | 205 | 84.1 | 4.97 |
| Lin [17] | 43.7 | 7.5 | 90 | 25 | - | 3.1 | 0.3 | 0.3 | 152 | 38 | 205 | 86.2 | 5.15 |
| Lin [17] | 43.7 | 7.5 | 120 | 25 | - | 3.1 | 0.3 | 0.3 | 152 | 38 | 205 | 86.9 | 5.19 |
| Lin [17] | 43.7 | 7.5 | 180 | 25 | - | 3.1 | 0.3 | 0.3 | 152 | 38 | 205 | 87.1 | 5.22 |
| Lin [17] | 43.7 | 7.5 | 365 | 25 | - | 3.1 | 0.3 | 0.3 | 152 | 38 | 205 | 88.1 | 5.23 |
| Lin [17] | 43.7 | 7.5 | 3 | 25 | - | 3.1 | 0.32 | 0.457 | 152 | 39 | 208 | 44 | 3.16 |
| Lin [17] | 43.7 | 7.5 | 7 | 25 | - | 3.1 | 0.32 | 0.457 | 152 | 39 | 208 | 56.1 | 3.54 |
| Lin [17] | 43.7 | 7.5 | 28 | 25 | - | 3.1 | 0.32 | 0.457 | 152 | 39 | 208 | 70.7 | 4.32 |
| Lin [17] | 43.7 | 7.5 | 60 | 25 | - | 3.1 | 0.32 | 0.457 | 152 | 39 | 208 | 78.7 | 4.76 |
| Lin [17] | 43.7 | 7.5 | 90 | 25 | - | 3.1 | 0.32 | 0.457 | 152 | 39 | 208 | 82.5 | 4.96 |
| Lin [17] | 43.7 | 7.5 | 120 | 25 | - | 3.1 | 0.32 | 0.457 | 152 | 39 | 208 | 85.7 | 5.11 |
| Lin [17] | 43.7 | 7.5 | 180 | 25 | - | 3.1 | 0.32 | 0.457 | 152 | 39 | 208 | 88.2 | 5.25 |
| Lin [17] | 43.7 | 7.5 | 365 | 25 | - | 3.1 | 0.32 | 0.457 | 152 | 39 | 208 | 92 | 5.40 |
| Wang [18] | 55.8 | 10.8 | 7 | - | 4.2 | 2.8 | 0.24 | 0.3 | 135 | 34 | 180 | 76.7 | 6.0 |
| Wang [18] | 55.8 | 10.8 | 28 | - | 4.2 | 2.8 | 0.24 | 0.3 | 135 | 34 | 180 | 93.5 | 6.7 |
| Wang [18] | 55.8 | 10.8 | 7 | - | 9.69 | 2.74 | 0.24 | 0.3 | 135 | 34 | 180 | 84.4 | 5.8 |
| Wang [18] | 55.8 | 10.8 | 28 | - | 9.69 | 2.74 | 0.24 | 0.3 | 135 | 34 | 180 | 95.3 | 6.9 |
| Wang [18] | 55.8 | 10.8 | 7 | - | 4.2 | 2.8 | 0.24 | 0.3 | 135 | 34 | 180 | 78.1 | 5.5 |
| Wang [18] | 55.8 | 10.8 | 28 | - | 4.2 | 2.8 | 0.24 | 0.3 | 135 | 34 | 180 | 90.2 | 6.2 |
| Wang [18] | 55.8 | 10.8 | 7 | - | 4.2 | 2.8 | 0.24 | 0.3 | 135 | 34 | 180 | 82.4 | 5.8 |
| Wang [18] | 55.8 | 10.8 | 28 | - | 4.2 | 2.8 | 0.24 | 0.3 | 135 | 34 | 180 | 90.6 | 6.4 |
| Wang [18] | 55.8 | 10.8 | 7 | - | 9.69 | 2.74 | 0.24 | 0.3 | 135 | 34 | 180 | 83.1 | 5.9 |
| Wang [18] | 55.8 | 10.8 | 28 | - | 9.69 | 2.74 | 0.24 | 0.3 | 135 | 34 | 180 | 89.4 | 6.7 |
| Yang [19] | 55.2 | 8.9 | 28 | 25 | 0.8 | 2.85 | 0.33 | 0.47 | 176 | 41.7 | 240 | 67.3 | 4.35 |
| Yang [19] | 55.2 | 8.9 | 28 | 25 | 0.8 | 2.85 | 0.33 | 0.47 | 168 | 38.8 | 220 | 67.4 | 4.38 |
| Yang [19] | 55.2 | 8.9 | 28 | 25 | 0.8 | 2.85 | 0.33 | 0.47 | 160 | 36.1 | 190 | 68.0 | 4.48 |
| Yang [19] | 55.2 | 8.9 | 28 | 25 | 0.8 | 2.85 | 0.33 | 0.47 | 153 | 33.5 | 30 | 69.1 | 4.52 |
| Yang [19] | 55.2 | 8.9 | 28 | 25 | 0.8 | 2.85 | 0.33 | 0.47 | 166 | 35 | 203 | 68.0 | 4.42 |
| Yang [19] | 55.2 | 8.9 | 28 | 25 | 0.8 | 2.85 | 0.33 | 0.47 | 160 | 36 | 190 | 68.0 | 4.48 |
| Yang [19] | 55.2 | 8.9 | 28 | 25 | 0.8 | 2.85 | 0.33 | 0.47 | 155 | 37 | 130 | 69.3 | 4.50 |
| Yang [19] | 55.2 | 8.9 | 28 | 25 | 0.8 | 2.85 | 0.33 | 0.47 | 149 | 38 | 60 | 73.5 | 4.55 |
| Yang [19] | 55.2 | 8.9 | 28 | 25 | 0.8 | 2.85 | 0.33 | 0.47 | 144 | 39 | 25 | 68.8 | 4.32 |
| Yang [19] | 55.2 | 8.9 | 28 | 25 | 0.8 | 2.85 | 0.33 | 0.47 | 153 | 33.5 | 140 | 69.1 | 4.52 |
| Yang [19] | 55.2 | 8.9 | 28 | 25 | 0.8 | 2.85 | 0.33 | 0.47 | 147 | 34.5 | 130 | 69.2 | 4.53 |
| Yang [19] | 55.2 | 8.9 | 28 | 25 | 0.8 | 2.85 | 0.33 | 0.47 | 145 | 35 | 115 | 70.8 | 4.55 |
| Yang [19] | 55.2 | 8.9 | 28 | 25 | 0.8 | 2.85 | 0.33 | 0.47 | 142 | 35.5 | 105 | 71.9 | 4.58 |
| Yang [19] | 55.2 | 8.9 | 28 | 25 | 0.8 | 2.85 | 0.33 | 0.47 | 139 | 36 | 85 | 70.3 | 4.43 |
| Yang [19] | 55.2 | 8.9 | 28 | 25 | 0.8 | 2.85 | 0.45 | 0.47 | 173 | 38 | 260 | 46.3 | 3.25 |
| Yang [19] | 55.2 | 8.9 | 28 | 25 | 0.8 | 2.85 | 0.40 | 0.47 | 164 | 38 | 240 | 55.0 | 3.56 |
| Yang [19] | 55.2 | 8.9 | 28 | 25 | 0.8 | 2.85 | 0.35 | 0.47 | 154 | 38 | 160 | 66.5 | 3.85 |
| Yang [19] | 55.2 | 8.9 | 28 | 25 | 0.8 | 2.85 | 0.33 | 0.47 | 149 | 38 | 60 | 73.5 | 4.55 |
| Yang [19] | 55.2 | 8.9 | 28 | 25 | 0.8 | 2.85 | 0.32 | 0.47 | 147 | 38 | 40 | 74.3 | 4.60 |
| Yang [19] | 55.2 | 8.9 | 28 | 25 | 0.8 | 2.85 | 0.30 | 0.47 | 142 | 38 | 80 | 82.5 | 4.65 |
| Yang [19] | 55.2 | 8.9 | 28 | 25 | 0.8 | 2.85 | 0.25 | 0.47 | 128 | 38 | 30 | 70.8 | 4.28 |
| Yang [19] | 55.2 | 8.9 | 28 | 25 | 0.8 | 2.85 | 0.3 | 0.43 | 120 | 38 | 50 | 78 | 4.35 |
| Yang [19] | 55.2 | 8.9 | 28 | 25 | 0.8 | 2.85 | 0.3 | 0.43 | 142 | 38 | 50 | 82.5 | 4.65 |
| Yang [19] | 55.2 | 8.9 | 28 | 25 | 0.8 | 2.85 | 0.3 | 0.43 | 163 | 38 | 50 | 76.5 | 4.25 |
| Yang [19] | 55.2 | 8.9 | 28 | 25 | 0.8 | 2.85 | 0.3 | 0.43 | 184 | 38 | 50 | 76 | 4.19 |
| Yang [19] | 55.2 | 8.9 | 28 | 25 | 0.8 | 2.85 | 0.3 | 0.43 | 206 | 38 | 50 | 74.2 | 4.14 |
| Yang [19] | 55.2 | 8.9 | 28 | 25 | 0.8 | 2.85 | 0.3 | 0.43 | 248 | 38 | 50 | 74 | 4.13 |
| Yang [19] | 55.2 | 8.9 | 28 | 25 | 0.8 | 2.85 | 0.3 | 0.43 | 291 | 38 | 50 | 74 | 4.12 |
| He and Yao [20] |  |  | 28 | 20 | - | 3.0 | 0.44 | 0.51 | 205 | 39 | 195 | 52.03 | 3.3 |
| He and Yao [20] |  |  | 28 | 20 | - | 3.0 | 0.39 | 0.47 | 205 | 38 | 195 | 60.14 | 3.7 |
| He and Yao [20] |  |  | 28 | 20 | - | 3.0 | 0.33 | 0.41 | 170 | 38 | 225 | 80.37 | 4.4 |
| He and Yao [20] |  |  | 28 | 20 | - | 3.0 | 0.26 | 0.40 | 175 | 37 | 230 | 89.24 | 4.6 |
| Wang [21] | 52.6 | 9.4 | 7 | 25 | 0 | 3.1 | 0.55 | 0.55 | 180 | 42 | 120 | 24.3 | 1.28 |
| Wang [21] | 52.6 | 9.4 | 28 | 25 | 0 | 3.1 | 0.55 | 0.55 | 180 | 42 | 120 | 34.6 | 1.68 |
| Wang [21] | 52.6 | 9.4 | 7 | 25 | 3 | 3.1 | 0.55 | 0.55 | 180 | 42 | 140 | 24.8 | 1.34 |
| Wang [21] | 52.6 | 9.4 | 28 | 25 | 3 | 3.1 | 0.55 | 0.55 | 180 | 42 | 140 | 34.9 | 1.80 |
| Wang [21] | 52.6 | 9.4 | 7 | 25 | 5 | 3.1 | 0.55 | 0.55 | 180 | 42 | 150 | 25.9 | 1.34 |
| Wang [21] | 52.6 | 9.4 | 28 | 25 | 5 | 3.1 | 0.55 | 0.55 | 180 | 42 | 150 | 35.9 | 1.82 |
| Wang [21] | 52.6 | 9.4 | 7 | 25 | 7 | 3.1 | 0.55 | 0.55 | 180 | 42 | 175 | 25.5 | 1.48 |
| Wang [21] | 52.6 | 9.4 | 28 | 25 | 7 | 3.1 | 0.55 | 0.55 | 180 | 42 | 175 | 36.4 | 1.96 |
| Wang [21] | 52.6 | 9.4 | 7 | 25 | 10 | 3.1 | 0.55 | 0.55 | 180 | 42 | 180 | 26.3 | 1.64 |
| Wang [21] | 52.6 | 9.4 | 28 | 25 | 10 | 3.1 | 0.55 | 0.55 | 180 | 42 | 180 | 37.9 | 2.15 |
| Wang [21] | 52.6 | 9.4 | 7 | 25 | 15 | 3.1 | 0.55 | 0.55 | 180 | 42 | 190 | 27.4 | 1.72 |
| Wang [21] | 52.6 | 9.4 | 28 | 25 | 15 | 3.1 | 0.55 | 0.55 | 180 | 42 | 190 | 38.2 | 2.19 |
| Wang [21] | 52.6 | 9.4 | 7 | 25 | 20 | 3.1 | 0.55 | 0.55 | 180 | 42 | 160 | 25.7 | 1.81 |
| Wang [21] | 52.6 | 9.4 | 28 | 25 | 20 | 3.1 | 0.55 | 0.55 | 180 | 42 | 160 | 36.5 | 2.17 |
| Cao and Yang [22] | - | - | 3 | 20 | 3 | 3.3 | 0.30 | 0.38 | 166 | 48 | 210 | 48.9 | 3.35 |
| Cao and Yang [22] | - | - | 7 | 20 | 3 | 3.3 | 0.30 | 0.38 | 166 | 48 | 210 | 64.1 | 5.36 |
| Cao and Yang [22] | - | - | 28 | 20 | 3 | 3.3 | 0.30 | 0.38 | 166 | 48 | 210 | 80.2 | 5.75 |
| Cao and Yang [22] | - | - | 3 | 20 | 7 | 3.3 | 0.30 | 0.38 | 166 | 48 | 210 | 49.0 | 3.07 |
| Cao and Yang [22] | - | - | 7 | 20 | 7 | 3.3 | 0.30 | 0.38 | 166 | 48 | 210 | 66.3 | 5.65 |
| Cao and Yang [22] | - | - | 28 | 20 | 7 | 3.3 | 0.30 | 0.38 | 166 | 48 | 210 | 80.1 | 6.01 |
| Cao and Yang [22] | - | - | 3 | 20 | 10 | 3.3 | 0.30 | 0.38 | 166 | 48 | 220 | 48.2 | 2.97 |
| Cao and Yang [22] | - | - | 7 | 20 | 10 | 3.3 | 0.30 | 0.38 | 166 | 48 | 220 | 61.7 | 5.33 |
| Cao and Yang [22] | - | - | 28 | 20 | 10 | 3.3 | 0.30 | 0.38 | 166 | 48 | 220 | 75.3 | 5.85 |
| Wu et al. [23] | - | - | 28 | - | - | - | 0.44 | 0.44 | - | 30 | - | 51.2 | 3.78 |
| Wu et al. [23] | - | - | 28 | - | - | - | 0.44 | 0.44 | - | 30 | - | 54 | 3.96 |
| Li [24] | 44.5 | 7.8 | 28 | 25 | 4.2 | 2.6 | 0.54 | 0.54 | 185 | 38 | 40 | 28.8 | 2.73 |
| Li [24] | 44.5 | 7.8 | 28 | 25 | 4.2 | 2.6 | 0.45 | 0.45 | 185 | 38 | 45 | 35 | 3.31 |
| Li [24] | 44.5 | 7.8 | 28 | 25 | 4.2 | 2.6 | 0.4 | 0.4 | 190 | 38 | 45 | 39.8 | 3.72 |
| Tan [25] | - | - | 28 | 80 | 18 | 2.8 | 0.46 | 0.46 | 135 | 27 | - | 28.9 | 1.98 |
| Tan [25] | - | - | 28 | 80 | 16.8 | 2.8 | 0.46 | 0.46 | 160 | 32 | - | 33.3 | 2.42 |
| Tan [25] | - | - | 28 | 80 | 21.7 | 2.8 | 0.46 | 0.46 | 160 | 32 | - | 30.2 | 2.26 |
| Tan [25] | - | - | 28 | 80 | 21.8 | 2.8 | 0.48 | 0.48 | 150 | 34 | - | 30 | 2.15 |
| Tan [25] | - | - | 28 | 80 | 21.8 | 2.8 | 0.48 | 0.48 | 130 | 28 | - | 28 | 1.98 |
| Tan [25] | - | - | 28 | 80 | 16.7 | 2.8 | 0.48 | 0.48 | 130 | 28 | - | 25.3 | 1.89 |
| Tan [25] | - | - | 28 | 80 | 18.0 | 2.8 | 0.53 | 0.53 | 130 | 29 | - | 24.9 | 1.84 |
| Tan [25] | - | - | 28 | 80 | 18.2 | 2.8 | 0.53 | 0.53 | 130 | 29 | - | 22.5 | 1.7 |
| Tan [25] | - | - | 28 | 80 | 17.4 | 2.8 | 0.53 | 0.53 | 130 | 29 | - | 26.7 | 1.84 |
| Tan [25] | - | - | 28 | 80 | 17.5 | 2.8 | 0.58 | 0.58 | 134 | 29 | - | 24.4 | 1.7 |
| Tan [25] | - | - | 28 | 80 | 16.8 | 2.8 | 0.58 | 0.58 | 134 | 29 | - | 29.6 | 2.25 |
| Tan [25] | - | - | 28 | 80 | 20.0 | 2.8 | 0.58 | 0.58 | 134 | 29 | - | 21.3 | 1.7 |
| Tan [25] | - | - | 28 | 80 | 18.2 | 2.8 | 0.63 | 0.63 | 133 | 30 | - | 20 | 1.59 |
| Tan [25] | - | - | 28 | 80 | 18.2 | 2.8 | 0.63 | 0.63 | 138 | 30 | - | 19 | 1.36 |
| Tan [25] | - | - | 28 | 80 | 20.0 | 2.8 | 0.63 | 0.63 | 133 | 31 | - | 18.2 | 1.42 |
| Huang and Han [26] | - | - | 28 | 20 | 12.1* | 3.1 | 0.5 | 0.5 | - | - | 72 | 26.5 | 2.69 |
| Huang and Han [26] | - | - | 28 | 20 | 15.0* | 3.1 | 0.5 | 0.5 | - | - | 57 | 31.8 | 2.91 |
| Huang and Han [26] | - | - | 28 | 20 | 18.0* | 3.1 | 0.5 | 0.5 | - | - | 60 | 30.9 | 2.83 |
| Huang and Han [26] | - | - | 28 | 20 | 21.0* | 3.1 | 0.5 | 0.5 | - | - | 58 | 30.8 | 2.69 |
| Huang and Han [26] | - | - | 28 | - | 13.8* | 2.6 | 0.5 | 0.5 | - | - | 60 | 22.3 | 2.26 |
| Huang and Han [26] | - | - | 28 | - | 20.8* | 2.9 | 0.5 | 0.5 | - | - | 60 | 28.4 | 2.42 |
| Huang and Han [26] | - | - | 28 | - | 25.9* | 3.3 | 0.5 | 0.5 | - | - | 58 | 26.5 | 2.36 |
| Huang and Han [26] | - | - | 28 | 40 | 5* | - | 0.5 | 0.5 | 187 | - | - | 27.5 | 2.31 |
| Huang and Han [26] | - | - | 28 | 40 | 10* | - | 0.5 | 0.5 | 188 | - | - | 28.1 | 2.42 |
| Huang and Han [26] | - | - | 28 | 40 | 15* | - | 0.5 | 0.5 | 189 | - | - | 29.2 | 2.39 |
| Huang and Han [26] | - | - | 28 | 40 | 20* | - | 0.5 | 0.5 | 190 | - | - | 28.7 | 2.54 |
| Huang and Han [26] | - | - | 28 | 40 | 25* | - | 0.5 | 0.5 | 191 | - | - | 29.8 | 2.36 |
| Huang and Han [26] | - | - | 28 | 40 | 5* | - | 0.6 | 0.6 | 183 | - | - | 24.2 | 2 |
| Huang and Han [26] | - | - | 28 | 40 | 10* | - | 0.6 | 0.6 | 184 | - | - | 25.1 | 2.04 |
| Huang and Han [26] | - | - | 28 | 40 | 15* | - | 0.6 | 0.6 | 185 | - | - | 26.6 | 2.31 |
| Huang and Han [26] | - | - | 28 | 40 | 20* | - | 0.6 | 0.6 | 186 | - | - | 23.5 | 2.17 |
| Huang and Han [26] | - | - | 28 | 40 | 25* | - | 0.6 | 0.6 | 187 | - | - | 23.1 | 1.99 |
| Huang and Han [26] | - | - | 28 | 80 | 5* | - | 0.5 | 0.5 | 150 | - | - | 29 | 2.5 |
| Huang and Han [26] | - | - | 28 | 80 | 10* | - | 0.5 | 0.5 | 151 | - | - | 31.2 | 2.64 |
| Huang and Han [26] | - | - | 28 | 80 | 15* | - | 0.5 | 0.5 | 152 | - | - | 34.8 | 2.76 |
| Huang and Han [26] | - | - | 28 | 80 | 20* | - | 0.5 | 0.5 | 153 | - | - | 33.5 | 2.57 |
| Huang and Han [26] | - | - | 28 | 80 | 25* | - | 0.5 | 0.5 | 154 | - | - | 32.9 | 2.49 |
| Huang and Han [26] | - | - | 28 | 80 | 5* | - | 0.7 | 0.7 | 151 | - | - | 21.6 | 1.87 |
| Huang and Han [26] | - | - | 28 | 80 | 10* | - | 0.7 | 0.7 | 151 | - | - | 23.3 | 2.09 |
| Huang and Han [26] | - | - | 28 | 80 | 15* | - | 0.7 | 0.7 | 152 | - | - | 26.3 | 2.09 |
| Huang and Han [26] | - | - | 28 | 80 | 20* | - | 0.7 | 0.7 | 152 | - | - | 23.5 | 1.74 |
| Huang and Han [26] | - | - | 28 | 80 | 25* | - | 0.7 | 0.7 | 154 | - | - | 22.5 | 1.7 |
| Cai [27] | 44.8 | 7.9 | 7 | 31.5 | 5 | 2.6 | 0.5 | 0.5 | 180 | 42 | 200 | 25.9 | 1.38 |
| Cai [27] | 44.8 | 7.9 | 28 | 31.5 | 5 | 2.6 | 0.5 | 0.5 | 180 | 42 | 200 | 35.9 | 1.82 |
| Cai [27] | 44.8 | 7.9 | 7 | 31.5 | 10 | 2.6 | 0.5 | 0.5 | 180 | 42 | 200 | 25.5 | 1.64 |
| Cai [27] | 44.8 | 7.9 | 28 | 31.5 | 10 | 2.6 | 0.5 | 0.5 | 180 | 42 | 200 | 35.9 | 2.15 |
| Cai [27] | 44.8 | 7.9 | 7 | 31.5 | 15 | 2.6 | 0.5 | 0.5 | 180 | 42 | 210 | 25.4 | 1.72 |
| Cai [27] | 44.8 | 7.9 | 28 | 31.5 | 15 | 2.6 | 0.5 | 0.5 | 180 | 42 | 210 | 35.9 | 2.19 |
| Cai [27] | 44.8 | 7.9 | 7 | 31.5 | 20 | 2.6 | 0.5 | 0.5 | 180 | 42 | 220 | 24.7 | 2.01 |
| Cai [27] | 44.8 | 7.9 | 28 | 31.5 | 20 | 2.6 | 0.5 | 0.5 | 180 | 42 | 220 | 35.5 | 2.17 |
| Liu et al. [28] | 47.6 | 8.5 | 7 | 40 | 6* | 3.2 | 0.55 | 0.55 | 175 | 42 | 100 | 27.3 | 1.6 |
| Liu et al. [28] | 47.6 | 8.5 | 7 | 40 | 12* | 3.2 | 0.55 | 0.55 | 175 | 41 | 85 | 30.1 | 1.7 |
| Liu et al. [28] | 47.6 | 8.5 | 7 | 40 | 18* | 3.2 | 0.55 | 0.55 | 175 | 40 | 75 | 31.7 | 2.0 |
| Liu et al. [28] | 47.6 | 8.5 | 28 | 40 | 6* | 3.2 | 0.55 | 0.55 | 175 | 42 | 100 | 33.3 | 2.1 |
| Liu et al. [28] | 47.6 | 8.5 | 28 | 40 | 12* | 3.2 | 0.55 | 0.55 | 175 | 41 | 85 | 36.3 | 2.4 |
| Liu et al. [28] | 47.6 | 8.5 | 28 | 40 | 18* | 3.2 | 0.55 | 0.55 | 175 | 40 | 75 | 38.0 | 2.6 |
| Qin [29] | 51.7 | 9.1 | 28 | 25 | 6 | 3.2 | 0.38 | 0.38 | 179 | 35 | 180 | 61.3 | 4.25 |
| Qin [29] | 51.7 | 9.1 | 28 | 25 | 9 | 3.2 | 0.38 | 0.38 | 179 | 35 | 180 | 62.7 | 4.34 |
| Qin [29] | 51.7 | 9.1 | 28 | 25 | 12 | 3.2 | 0.38 | 0.38 | 179 | 35 | 180 | 60.8 | 4.01 |
| Qin [29] | 51.7 | 9.1 | 28 | 25 | 12* | 3.2 | 0.38 | 0.38 | 179 | 35 | 140 | 60.6 | 4.29 |
| Qin [29] | 51.7 | 9.1 | 28 | 25 | 12* | 3.2 | 0.38 | 0.38 | 179 | 35 | 155 | 62.4 | 4.17 |
| Wang and Chen [30] | 60.8 | 8.5 | 28 | 80 | - | 2.67 | 0.38 | 0.38 | 114 | 26 | - | 39.8 | 2.71 |
| Wang and Chen [30] | 60.8 | 8.5 | 28 | 80 | - | 2.67 | 0.38 | 0.38 | 114 | 26 | - | 34.3 | 2.92 |
| Wang and Chen [30] | 47.7 | 7.9 | 28 | 80 | - | 2.67 | 0.62 | 0.77 | 120 | 30 | - | 17.9 | 1.22 |
| Wang and Chen [30] | 47.7 | 7.9 | 28 | 80 | - | 2.67 | 0.62 | 0.77 | 120 | 30 | - | 18.6 | 1.80 |
| Wang and Chen [30] | 47.7 | 7.9 | 28 | 80 | - | 2.67 | 0.62 | 0.77 | 120 | 30 | - | 20.6 | 1.94 |
| Wang and Chen [30] | 47.7 | 7.9 | 28 | 80 | - | 2.67 | 0.62 | 0.77 | 120 | 30 | - | 18.2 | 1.80 |
| Wang and Chen [30] | 47.7 | 7.9 | 28 | 80 | - | 2.67 | 0.62 | 0.77 | 120 | 30 | - | 23.7 | 1.83 |
| Chen [31] | - | - | 28 | 80 | 12* | 2.88 | 0.50 | 0.625 | 110 | 26 | 61 | 32.5 | 2.88 |
| Chen [31] | - | - | 28 | 80 | 16* | 2.73 | 0.50 | 0.625 | 110 | 26 | 51 | 35.8 | 2.73 |
| Chen [31] | - | - | 28 | 80 | 16* | 2.73 | 0.50 | 0.625 | 110 | 28 | 52 | 30.2 | 2.40 |
| Chen [31] | - | - | 28 | 80 | 21* | 2.54 | 0.40 | 0.5 | 110 | 24 | 56 | 39.6 | 2.74 |
| Chen [31] | - | - | 28 | 80 | 21* | 2.54 | 0.50 | 0.625 | 110 | 26 | 58 | 30.7 | 2.47 |
| Chen [31] | - | - | 28 | 80 | 21* | 2.54 | 0.60 | 0.75 | 110 | 28 | 53 | 36.5 | 2.14 |
| Wang et al. [32] | - | - | 7 | - | 0* | - | 0.4 | 0.4 | 190 | 30 | <10 | 37.2 | 2.45 |
| Wang et al. [32] | - | - | 7 | - | 10* | - | 0.4 | 0.4 | 190 | 30 | <10 | 29.6 | 2.69 |
| Wang et al. [32] | - | - | 7 | - | 20* | - | 0.4 | 0.4 | 190 | 30 | <10 | 38.4 | 2.40 |
| Wang et al. [32] | - | - | 7 | - | 30* | - | 0.4 | 0.4 | 190 | 30 | <10 | 41.1 | 2.78 |
| Wang et al. [32] | - | - | 7 | - | 40* | - | 0.4 | 0.4 | 190 | 30 | <10 | 42.5 | 3.07 |
| Wang et al. [32] | - | - | 7 | - | 0* | - | 0.7 | 0.7 | 190 | 34 | <10 | 16.4 | 1.13 |
| Wang et al. [32] | - | - | 7 | - | 10* | - | 0.7 | 0.7 | 190 | 34 | <10 | 17.9 | 1.36 |
| Wang et al. [32] | - | - | 7 | - | 20* | - | 0.7 | 0.7 | 190 | 34 | <10 | 16.3 | 1.08 |
| Wang et al. [32] | - | - | 7 | - | 30* | - | 0.7 | 0.7 | 190 | 34 | <10 | 19.7 | 1.47 |
| Wang et al. [32] | - | - | 7 | - | 40* | - | 0.7 | 0.7 | 190 | 34 | <10 | 21.0 | 1.38 |
| Wang et al. [32] | - | - | 7 | - | 0* | - | 1.0 | 1.0 | 190 | 42 | <10 | 7.9 | 0.62 |
| Wang et al. [32] | - | - | 7 | - | 10* | - | 1.0 | 1.0 | 190 | 42 | <10 | 9.5 | 0.71 |
| Wang et al. [32] | - | - | 7 | - | 20* | - | 1.0 | 1.0 | 190 | 42 | <10 | 10.5 | 0.80 |
| Wang et al. [32] | - | - | 7 | - | 30* | - | 1.0 | 1.0 | 190 | 42 | <10 | 12.4 | 0.99 |
| Wang et al. [32] | - | - | 7 | - | 40* | - | 1.0 | 1.0 | 190 | 42 | <10 | 13.1 | 1.11 |
| Wang et al. [32] | - | - | 28 | - | 0* | - | 0.4 | 0.4 | 190 | 30 | <10 | 46.4 | 2.88 |
| Wang et al. [32] | - | - | 28 | - | 10* | - | 0.4 | 0.4 | 190 | 30 | <10 | 48.1 | 3.01 |
| Wang et al. [32] | - | - | 28 | - | 20* | - | 0.4 | 0.4 | 190 | 30 | <10 | 50.5 | 3.23 |
| Wang et al. [32] | - | - | 28 | - | 30* | - | 0.4 | 0.4 | 190 | 30 | <10 | 50.1 | 2.90 |
| Wang et al. [32] | - | - | 28 | - | 40* | - | 0.4 | 0.4 | 190 | 30 | <10 | 51.6 | 3.15 |
| Wang et al. [32] | - | - | 28 | - | 0* | - | 0.7 | 0.7 | 190 | 34 | <10 | 23.8 | 1.82 |
| Wang et al. [32] | - | - | 28 | - | 10* | - | 0.7 | 0.7 | 190 | 34 | <10 | 24.4 | 1.90 |
| Wang et al. [32] | - | - | 28 | - | 20* | - | 0.7 | 0.7 | 190 | 34 | <10 | 27.7 | 1.62 |
| Wang et al. [32] | - | - | 28 | - | 30* | - | 0.7 | 0.7 | 190 | 34 | <10 | 25.7 | 1.86 |
| Wang et al. [32] | - | - | 28 | - | 40* | - | 0.7 | 0.7 | 190 | 34 | <10 | 26.7 | 1.58 |
| Wang et al. [32] | - | - | 28 | - | 0* | - | 1.0 | 1.0 | 190 | 42 | <10 | 14.1 | 0.98 |
| Wang et al. [32] | - | - | 28 | - | 10* | - | 1.0 | 1.0 | 190 | 42 | <10 | 13.0 | 1.18 |
| Wang et al. [32] | - | - | 28 | - | 20* | - | 1.0 | 1.0 | 190 | 42 | <10 | 14.2 | 1.23 |
| Wang et al. [32] | - | - | 28 | - | 30* | - | 1.0 | 1.0 | 190 | 42 | <10 | 17.2 | 1.33 |
| Wang et al. [32] | - | - | 28 | - | 40* | - | 1.0 | 1.0 | 190 | 42 | <10 | 17.3 | 1.39 |
| Xing et al.[33] | - | - | 7 | 80 | 15* | 2.8 | 0.5 | 0.66 | 104 | 30 | 44 | 20.4 | 1.61 |
| Xing et al.[33] | - | - | 7 | 80 | 15* | 2.8 | 0.5 | 0.66 | 108 | 30 | 49 | 19.9 | 1.57 |
| Xing et al.[33] | - | - | 7 | 80 | 15* | 2.8 | 0.5 | 0.66 | 110 | 30 | 36 | 19.2 | 1.53 |
| Xing et al.[33] | - | - | 7 | 80 | 15* | 2.8 | 0.5 | 0.66 | 114 | 30 | 45 | 18.4 | 1.50 |
| Xing et al.[33] | - | - | 7 | 80 | 15* | 2.8 | 0.5 | 0.66 | 115 | 30 | 40 | 18.4 | 1.50 |
| Xing et al.[33] | - | - | 28 | 80 | 15* | 2.8 | 0.5 | 0.66 | 104 | 30 | 44 | 29.7 | 3.04 |
| Xing et al.[33] | - | - | 28 | 80 | 15* | 2.8 | 0.5 | 0.66 | 108 | 30 | 49 | 29.7 | 3.02 |
| Xing et al.[33] | - | - | 28 | 80 | 15* | 2.8 | 0.5 | 0.66 | 110 | 30 | 36 | 28.5 | 2.91 |
| Xing et al.[33] | - | - | 28 | 80 | 15* | 2.8 | 0.5 | 0.66 | 114 | 30 | 45 | 27.4 | 2.84 |
| Xing et al.[33] | - | - | 28 | 80 | 15* | 2.8 | 0.5 | 0.66 | 115 | 30 | 40 | 27.1 | 2.75 |
| Xing et al.[33] | - | - | 90 | 80 | 15* | 2.8 | 0.5 | 0.66 | 104 | 30 | 44 | 40.9 | 3.43 |
| Xing et al.[33] | - | - | 90 | 80 | 15* | 2.8 | 0.5 | 0.66 | 108 | 30 | 49 | 40.2 | 3.37 |
| Xing et al.[33] | - | - | 90 | 80 | 15* | 2.8 | 0.5 | 0.66 | 110 | 30 | 36 | 39.4 | 3.26 |
| Xing et al.[33] | - | - | 90 | 80 | 15* | 2.8 | 0.5 | 0.66 | 114 | 30 | 45 | 38.3 | 3.19 |
| Xing et al.[33] | - | - | 90 | 80 | 15* | 2.8 | 0.5 | 0.66 | 115 | 30 | 40 | 37.5 | 3.15 |
| Hong et al.[34] | - | - | 28 | - | 24* | 2.7 | 0.57 | 0.57 | 192 | 35 | 86 | 21.8 | 2.41 |
| Hong et al.[34] | - | - | 28 | - | 24* | 2.7 | 0.43 | 0.43 | 193 | 32 | 80 | 39.7 | 3.68 |
| Li and Gou [35] | - | - | 7 | 120 | 11.2~13.0* | 2.44~2.68 | 0.55 | 0.92 | 83.5 | 26.5 | 55 | 8.1 | 0.56 |
| Li and Gou [35] | - | - | 7 | 120 | 11.2~13.0* | 2.44~2.68 | 0.55 | 0.92 | 83.5 | 26.5 | 42 | 9.7 | 0.68 |
| Li and Gou [35] | - | - | 7 | 120 | 11.2~13.0* | 2.44~2.68 | 0.55 | 0.92 | 83.5 | 27.0 | 55 | 9.9 | 0.72 |
| Li and Gou [35] | - | - | 7 | 120 | 11.2~13.0* | 2.44~2.68 | 0.55 | 0.92 | 83.5 | 27.0 | 49 | 10.7 | 0.80 |
| Li and Gou [35] | - | - | 7 | 120 | 11.2~13.0* | 2.44~2.68 | 0.45 | 0.64 | 98 | 29.5 | 60 | 15.2 | 1.20 |
| Li and Gou [35] | - | - | 7 | 120 | 11.2~13.0* | 2.44~2.68 | 0.45 | 0.64 | 98 | 29.5 | 46 | 16.7 | 1.21 |
| Li and Gou [35] | - | - | 7 | 120 | 11.2~13.0* | 2.44~2.68 | 0.45 | 0.64 | 98 | 30.0 | 54 | 19.2 | 1.38 |
| Li and Gou [35] | - | - | 7 | 120 | 11.2~13.0* | 2.44~2.68 | 0.45 | 0.64 | 98 | 30.0 | 51 | 19.6 | 1.31 |
| Li and Gou [35] | - | - | 28 | 120 | 11.2~13.0* | 2.44~2.68 | 0.55 | 0.92 | 83.5 | 26.5 | 55 | 12.6 | 0.95 |
| Li and Gou [35] | - | - | 28 | 120 | 11.2~13.0* | 2.44~2.68 | 0.55 | 0.92 | 83.5 | 26.5 | 42 | 14.7 | 1.12 |
| Li and Gou [35] | - | - | 28 | 120 | 11.2~13.0* | 2.44~2.68 | 0.55 | 0.92 | 83.5 | 27.0 | 55 | 16.4 | 1.39 |
| Li and Gou [35] | - | - | 28 | 120 | 11.2~13.0* | 2.44~2.68 | 0.55 | 0.92 | 83.5 | 27.0 | 49 | 16.6 | 1.41 |
| Li and Gou [35] | - | - | 28 | 80 | 11.2~13.0* | 2.44~2.68 | 0.45 | 0.64 | 98 | 29.5 | 60 | 23.5 | 1.73 |
| Li and Gou [35] | - | - | 28 | 80 | 11.2~13.0* | 2.44~2.68 | 0.45 | 0.64 | 98 | 29.5 | 46 | 26.7 | 1.78 |
| Li and Gou [35] | - | - | 28 | 80 | 11.2~13.0* | 2.44~2.68 | 0.45 | 0.64 | 98 | 30.0 | 54 | 28.3 | 1.88 |
| Li and Gou [35] | - | - | 28 | 80 | 11.2~13.0* | 2.44~2.68 | 0.45 | 0.64 | 98 | 30.0 | 51 | 31.1 | 2.08 |
| Li and Gou [35] | - | - | 90 | 120 | 11.2~13.0* | 2.44~2.68 | 0.55 | 0.92 | 83.5 | 26.5 | 55 | 21.1 | 1.51 |
| Li and Gou [35] | - | - | 90 | 120 | 11.2~13.0* | 2.44~2.68 | 0.55 | 0.92 | 83.5 | 26.5 | 42 | 24.2 | 1.65 |
| Li and Gou [35] | - | - | 90 | 120 | 11.2~13.0* | 2.44~2.68 | 0.55 | 0.92 | 83.5 | 27.0 | 55 | 26.8 | 1.76 |
| Li and Gou [35] | - | - | 90 | 120 | 11.2~13.0* | 2.44~2.68 | 0.55 | 0.92 | 83.5 | 27.0 | 49 | 26.1 | 2.11 |
| Li and Gou [35] | - | - | 90 | 120 | 11.2~13.0* | 2.44~2.68 | 0.45 | 0.64 | 98 | 29.5 | 60 | 36.7 | 2.45 |
| Li and Gou [35] | - | - | 90 | 120 | 11.2~13.0* | 2.44~2.68 | 0.45 | 0.64 | 98 | 29.5 | 46 | 37.0 | 2.44 |
| Li and Gou [35] | - | - | 90 | 120 | 11.2~13.0* | 2.44~2.68 | 0.45 | 0.64 | 98 | 30.0 | 54 | 40.4 | 2.65 |
| Li and Gou [35] | - | - | 90 | 120 | 11.2~13.0* | 2.44~2.68 | 0.45 | 0.64 | 98 | 30.0 | 51 | 41.1 | 2.90 |
| Li et al. [36] | - | - | 7 | - | 11.2* | 2.44 | 0.45 | 0.64 | 87 | 27 | 57 | 18.6 | 1.43 |
| Li et al. [36] | - | - | 7 | - | 11.2* | 2.44 | 0.45 | 0.64 | 87 | 26 | 47 | 12.9 | 1.03 |
| Li et al. [36] | - | - | 7 | - | 11.2* | 2.44 | 0.50 | 0.83 | 87 | 28 | 52 | 13.3 | 0.98 |
| Li et al. [36] | - | - | 7 | - | 11.2* | 2.44 | 0.50 | 0.83 | 87 | 27 | 42 | 9.6 | 0.71 |
| Li et al. [36] | - | - | 7 | 40 | 13.0* | 2.68 | 0.50 | 0.83 | 89 | 36 | <10 | 15.5 | 0.79 |
| Li et al. [36] | - | - | 7 | 40 | 13.0* | 2.68 | 0.50 | 1.00 | 88 | 36 | <10 | 11.9 | 0.72 |
| Li et al. [36] | - | - | 7 | 40 | 13.0* | 2.68 | 0.50 | 1.11 | 88 | 36 | <10 | 9.3 | 0.61 |
| Li et al. [36] | - | - | 7 | 40 | 11.2* | 2.44 | 0.50 | 0.83 | 103 | 36 | <10 | 11.6 | 0.90 |
| Li et al. [36] | - | - | 7 | 40 | 11.2* | 2.44 | 0.50 | 1.00 | 102 | 36 | <10 | 9.2 | 0.72 |
| Li et al. [36] | - | - | 7 | 40 | 11.2* | 2.44 | 0.50 | 1.11 | 102 | 36 | <10 | 7.8 | 0.65 |
| Li et al. [36] | - | - | 28 | - | 11.2* | 2.44 | 0.45 | 0.64 | 87 | 27 | 57 | 29.8 | 2.23 |
| Li et al. [36] | - | - | 28 | - | 11.2* | 2.44 | 0.45 | 0.64 | 87 | 26 | 47 | 22.3 | 1.56 |
| Li et al. [36] | - | - | 28 | - | 11.2* | 2.44 | 0.50 | 0.83 | 87 | 28 | 52 | 23.5 | 1.73 |
| Li et al. [36] | - | - | 28 | - | 11.2* | 2.44 | 0.50 | 0.83 | 87 | 27 | 42 | 18.0 | 1.46 |
| Li et al. [36] | - | - | 28 | 40 | 13.0* | 2.68 | 0.50 | 0.83 | 89 | 36 | <10 | 23.6 | 2.04 |
| Li et al. [36] | - | - | 28 | 40 | 13.0* | 2.68 | 0.50 | 1.00 | 88 | 36 | <10 | 21.5 | 1.80 |
| Li et al. [36] | - | - | 28 | 40 | 13.0* | 2.68 | 0.50 | 1.11 | 88 | 36 | <10 | 19.9 | 1.62 |
| Li et al. [36] | - | - | 28 | 40 | 11.2* | 2.44 | 0.50 | 0.83 | 103 | 36 | <10 | 21.6 | 1.96 |
| Li et al. [36] | - | - | 28 | 40 | 11.2* | 2.44 | 0.50 | 1.00 | 102 | 36 | <10 | 20.0 | 1.69 |
| Li et al. [36] | - | - | 28 | 40 | 11.2* | 2.44 | 0.50 | 1.11 | 102 | 36 | <10 | 18.5 | 1.53 |
| Li et al. [36] | - | - | 90 | 40 | 13.0* | 2.68 | 0.50 | 0.83 | 89 | 36 | <10 | 32.8 | 2.20 |
| Li et al. [36] | - | - | 90 | 40 | 13.0* | 2.68 | 0.50 | 1.00 | 88 | 36 | <10 | 28.0 | 2.12 |
| Li et al. [36] | - | - | 90 | 40 | 13.0* | 2.68 | 0.50 | 1.11 | 88 | 36 | <10 | 25.5 | 2.10 |
| Li et al. [36] | - | - | 90 | 40 | 11.2* | 2.44 | 0.50 | 0.83 | 103 | 36 | <10 | 29.8 | 2.52 |
| Li et al. [36] | - | - | 90 | 40 | 11.2* | 2.44 | 0.50 | 1.00 | 102 | 36 | <10 | 27.8 | 2.18 |
| Li et al. [36] | - | - | 90 | 40 | 11.2* | 2.44 | 0.50 | 1.11 | 102 | 36 | <10 | 26.9 | 1.95 |
| Fu [37] | - | - | 28 | 80 | 10.8 | 2.42 | 0.45 | 0.64 | 98 | 30 | 33 | 28.8 | 1.71 |
| Fu [37] | - | - | 28 | 80 | 12.4 | 2.57 | 0.45 | 0.64 | 95 | 31 | 40 | 30.6 | 1.99 |
| Fu [37] | - | - | 28 | 120 | 10.8 | 2.42 | 0.55 | 0.92 | 88 | 27 | 42 | 14.7 | 1.10 |
| Fu [37] | - | - | 28 | 120 | 12.4 | 2.57 | 0.55 | 0.92 | 85 | 28 | 45 | 20.2 | 1.43 |
| Fu [37] | - | - | 90 | 80 | 10.8 | 2.42 | 0.45 | 0.64 | 98 | 30 | 33 | 41.3 | 2.33 |
| Fu [37] | - | - | 90 | 80 | 12.4 | 2.57 | 0.45 | 0.64 | 95 | 31 | 40 | 44.0 | 3.00 |
| Fu [37] | - | - | 90 | 120 | 10.8 | 2.42 | 0.55 | 0.92 | 88 | 27 | 42 | 24.8 | 1.80 |
| Fu [37] | - | - | 90 | 120 | 12.4 | 2.57 | 0.55 | 0.92 | 85 | 28 | 45 | 30.9 | 2.26 |
| Yang et al. [38] | 62.4 | 8.4 | 7 | 40 | 11.9* | 2.86 | 0.50 | 0.83 | 87~89 | - | <10 | 15.5 | 0.79 |
| Yang et al. [38] | 62.4 | 8.4 | 7 | 40 | 11.9* | 2.86 | 0.50 | 1.00 | 87~89 | - | <10 | 11.9 | 0.72 |
| Yang et al. [38] | 62.4 | 8.4 | 7 | 40 | 11.9* | 2.86 | 0.50 | 1.11 | 87~89 | - | <10 | 9.3 | 0.61 |
| Yang et al. [38] | 62.4 | 8.4 | 7 | 40 | 11.9* | 2.86 | 0.50 | 1.11 | 87~89 | - | <10 | 7.5 | 0.60 |
| Yang et al. [38] | 62.4 | 8.4 | 7 | 40 | 11.9* | 2.86 | 0.50 | 1.25 | 87~89 | - | <10 | 7.8 | 0.47 |
| Yang et al. [38] | 62.4 | 8.4 | 7 | 40 | 11.9* | 2.86 | 0.50 | 1.43 | 87~89 | - | <10 | 5.8 | 0.46 |
| Yang et al. [38] | 62.4 | 8.4 | 7 | 40 | 6.1* | 2.92 | 0.50 | 0.83 | 101~103 | - | <10 | 11.6 | 0.90 |
| Yang et al. [38] | 62.4 | 8.4 | 7 | 40 | 6.1* | 2.92 | 0.50 | 1.00 | 101~103 | - | <10 | 9.2 | 0.72 |
| Yang et al. [38] | 62.4 | 8.4 | 7 | 40 | 6.1* | 2.92 | 0.50 | 1.11 | 101~103 | - | <10 | 7.8 | 0.65 |
| Yang et al. [38] | 62.4 | 8.4 | 7 | 60 | 11.9* | 2.86 | 0.50 | 0.83 | 74~76 | - | <10 | 14.0 | 1.18 |
| Yang et al. [38] | 62.4 | 8.4 | 7 | 60 | 11.9* | 2.86 | 0.50 | 1.00 | 74~76 | - | <10 | 12.5 | 0.84 |
| Yang et al. [38] | 62.4 | 8.4 | 7 | 60 | 11.9* | 2.86 | 0.50 | 1.11 | 74~76 | - | <10 | 9.2 | 0.62 |
| Yang et al. [38] | 62.4 | 8.4 | 7 | 60 | 11.9* | 2.86 | 0.50 | 1.25 | 74~76 | - | <10 | 8.3 | 0.57 |
| Yang et al. [38] | 62.4 | 8.4 | 7 | 60 | 11.9* | 2.86 | 0.50 | 1.43 | 74~76 | - | <10 | 6.9 | 0.56 |
| Yang et al. [38] | 62.4 | 8.4 | 7 | 80 | 11.9* | 2.86 | 0.50 | 0.83 | 70~72 | - | <10 | 15.2 | 0.96 |
| Yang et al. [38] | 62.4 | 8.4 | 7 | 80 | 11.9* | 2.86 | 0.50 | 1.00 | 70~72 | - | <10 | 12.9 | 0.86 |
| Yang et al. [38] | 62.4 | 8.4 | 7 | 80 | 11.9* | 2.86 | 0.50 | 1.11 | 70~72 | - | <10 | 10.3 | 0.65 |
| Yang et al. [38] | 62.4 | 8.4 | 7 | 80 | 11.9* | 2.86 | 0.50 | 1.11 | 70~72 | - | <10 | 9.0 | 0.64 |
| Yang et al. [38] | 62.4 | 8.4 | 7 | 80 | 11.9* | 2.86 | 0.50 | 1.25 | 70~72 | - | <10 | 7.4 | 0.52 |
| Yang et al. [38] | 62.4 | 8.4 | 7 | 80 | 11.9* | 2.86 | 0.50 | 1.43 | 70~72 | - | <10 | 4.8 | 0.35 |
| Yang et al. [38] | 62.4 | 8.4 | 7 | 80 | 6.1* | 2.92 | 0.50 | 0.83 | 81~83 | - | <10 | 12.4 | 0.96 |
| Yang et al. [38] | 62.4 | 8.4 | 7 | 80 | 6.1* | 2.92 | 0.50 | 1.00 | 81~83 | - | <10 | 9.2 | 0.72 |
| Yang et al. [38] | 62.4 | 8.4 | 7 | 80 | 6.1* | 2.92 | 0.50 | 1.11 | 81~83 | - | <10 | 6.8 | 0.55 |
| Yang et al. [38] | 62.4 | 8.4 | 7 | 80 | 6.1* | 2.92 | 0.50 | 1.25 | 81~83 | - | <10 | 6.5 | 0.54 |
| Yang et al. [38] | 62.4 | 8.4 | 7 | 80 | 6.1* | 2.92 | 0.50 | 1.43 | 81~83 | - | <10 | 6.8 | 0.54 |
| Yang et al. [38] | 62.4 | 8.4 | 28 | 40 | 11.9* | 2.86 | 0.50 | 0.83 | 87~89 | - | <10 | 23.6 | 2.04 |
| Yang et al. [38] | 62.4 | 8.4 | 28 | 40 | 11.9* | 2.86 | 0.50 | 1.00 | 87~89 | - | <10 | 21.5 | 1.80 |
| Yang et al. [38] | 62.4 | 8.4 | 28 | 40 | 11.9* | 2.86 | 0.50 | 1.11 | 87~89 | - | <10 | 19.9 | 1.62 |
| Yang et al. [38] | 62.4 | 8.4 | 28 | 40 | 11.9* | 2.86 | 0.50 | 1.11 | 87~89 | - | <10 | 13.2 | 1.13 |
| Yang et al. [38] | 62.4 | 8.4 | 28 | 40 | 11.9* | 2.86 | 0.50 | 1.25 | 87~89 | - | <10 | 15.9 | 1.40 |
| Yang et al. [38] | 62.4 | 8.4 | 28 | 40 | 11.9* | 2.86 | 0.50 | 1.43 | 87~89 | - | <10 | 11.3 | 1.08 |
| Yang et al. [38] | 62.4 | 8.4 | 28 | 40 | 6.1* | 2.92 | 0.50 | 0.83 | 101~103 | - | <10 | 21.6 | 1.96 |
| Yang et al. [38] | 62.4 | 8.4 | 28 | 40 | 6.1* | 2.92 | 0.50 | 1.00 | 101~103 | - | <10 | 20.0 | 1.69 |
| Yang et al. [38] | 62.4 | 8.4 | 28 | 40 | 6.1* | 2.92 | 0.50 | 1.11 | 101~103 | - | <10 | 18.5 | 1.53 |
| Yang et al. [38] | 62.4 | 8.4 | 28 | 60 | 11.9* | 2.86 | 0.50 | 0.83 | 74~76 | - | <10 | 24.5 | 2.18 |
| Yang et al. [38] | 62.4 | 8.4 | 28 | 60 | 11.9* | 2.86 | 0.50 | 1.00 | 74~76 | - | <10 | 21.4 | 1.63 |
| Yang et al. [38] | 62.4 | 8.4 | 28 | 60 | 11.9* | 2.86 | 0.50 | 1.11 | 74~76 | - | <10 | 19.6 | 1.26 |
| Yang et al. [38] | 62.4 | 8.4 | 28 | 60 | 11.9* | 2.86 | 0.50 | 1.25 | 74~76 | - | <10 | 16.9 | 1.09 |
| Yang et al. [38] | 62.4 | 8.4 | 28 | 60 | 11.9* | 2.86 | 0.50 | 1.43 | 74~76 | - | <10 | 13.2 | 1.02 |
| Yang et al. [38] | 62.4 | 8.4 | 28 | 80 | 11.9* | 2.86 | 0.50 | 0.83 | 70~72 | - | <10 | 24.1 | 2.1 |
| Yang et al. [38] | 62.4 | 8.4 | 28 | 80 | 11.9* | 2.86 | 0.50 | 1.00 | 70~72 | - | <10 | 21.5 | 1.8 |
| Yang et al. [38] | 62.4 | 8.4 | 28 | 80 | 11.9* | 2.86 | 0.50 | 1.11 | 70~72 | - | <10 | 20.1 | 1.6 |
| Yang et al. [38] | 62.4 | 8.4 | 28 | 80 | 11.9* | 2.86 | 0.50 | 1.11 | 70~72 | - | <10 | 16.2 | 1.46 |
| Yang et al. [38] | 62.4 | 8.4 | 28 | 80 | 11.9* | 2.86 | 0.50 | 1.25 | 70~72 | - | <10 | 14.9 | 1.45 |
| Yang et al. [38] | 62.4 | 8.4 | 28 | 80 | 11.9* | 2.86 | 0.50 | 1.43 | 70~72 | - | <10 | 10.1 | 1.08 |
| Yang et al. [38] | 62.4 | 8.4 | 28 | 80 | 6.1* | 2.92 | 0.50 | 0.83 | 81~83 | - | <10 | 21.8 | 1.92 |
| Yang et al. [38] | 62.4 | 8.4 | 28 | 80 | 6.1* | 2.92 | 0.50 | 1.00 | 81~83 | - | <10 | 18.9 | 1.42 |
| Yang et al. [38] | 62.4 | 8.4 | 28 | 80 | 6.1* | 2.92 | 0.50 | 1.11 | 81~83 | - | <10 | 14.0 | 1.06 |
| Yang et al. [38] | 62.4 | 8.4 | 28 | 80 | 6.1* | 2.92 | 0.50 | 1.25 | 81~83 | - | <10 | 13.5 | 1.11 |
| Yang et al. [38] | 62.4 | 8.4 | 28 | 80 | 6.1* | 2.92 | 0.50 | 1.43 | 81~83 | - | <10 | 12.9 | 1.16 |
| Yang et al. [38] | 62.4 | 8.4 | 90 | 40 | 11.9* | 2.86 | 0.50 | 0.83 | 87~89 | - | <10 | 32.8 | 2.2 |
| Yang et al. [38] | 62.4 | 8.4 | 90 | 40 | 11.9* | 2.86 | 0.50 | 1.00 | 87~89 | - | <10 | 28.0 | 2.12 |
| Yang et al. [38] | 62.4 | 8.4 | 90 | 40 | 11.9* | 2.86 | 0.50 | 1.11 | 87~89 | - | <10 | 25.5 | 2.10 |
| Yang et al. [38] | 62.4 | 8.4 | 90 | 40 | 11.9* | 2.86 | 0.50 | 1.11 | 87~89 | - | <10 | 24.4 | 2.09 |
| Yang et al. [38] | 62.4 | 8.4 | 90 | 40 | 11.9* | 2.86 | 0.50 | 1.25 | 87~89 | - | <10 | 22.9 | 2.05 |
| Yang et al. [38] | 62.4 | 8.4 | 90 | 40 | 11.9* | 2.86 | 0.50 | 1.43 | 87~89 | - | <10 | 20.8 | 1.99 |
| Yang et al. [38] | 62.4 | 8.4 | 90 | 40 | 6.1* | 2.92 | 0.50 | 0.83 | 101~103 | - | <10 | 29.8 | 2.52 |
| Yang et al. [38] | 62.4 | 8.4 | 90 | 40 | 6.1* | 2.92 | 0.50 | 1.00 | 101~103 | - | <10 | 27.8 | 2.18 |
| Yang et al. [38] | 62.4 | 8.4 | 90 | 40 | 6.1* | 2.92 | 0.50 | 1.11 | 101~103 | - | <10 | 26.9 | 1.95 |
| Yang et al. [38] | 62.4 | 8.4 | 90 | 60 | 11.9* | 2.86 | 0.50 | 0.83 | 74~76 | - | <10 | 34.6 | 2.88 |
| Yang et al. [38] | 62.4 | 8.4 | 90 | 60 | 11.9* | 2.86 | 0.50 | 1.00 | 74~76 | - | <10 | 27.9 | 2.29 |
| Yang et al. [38] | 62.4 | 8.4 | 90 | 60 | 11.9* | 2.86 | 0.50 | 1.11 | 74~76 | - | <10 | 24.3 | 2.08 |
| Yang et al. [38] | 62.4 | 8.4 | 90 | 60 | 11.9* | 2.86 | 0.50 | 1.25 | 74~76 | - | <10 | 23.5 | 1.93 |
| Yang et al. [38] | 62.4 | 8.4 | 90 | 60 | 11.9* | 2.86 | 0.50 | 1.43 | 74~76 | - | <10 | 22.5 | 1.80 |
| Yang et al. [38] | 62.4 | 8.4 | 90 | 80 | 11.9* | 2.86 | 0.50 | 0.83 | 70~72 | - | <10 | 33.4 | 2.45 |
| Yang et al. [38] | 62.4 | 8.4 | 90 | 80 | 11.9* | 2.86 | 0.50 | 1.00 | 70~72 | - | <10 | 30.1 | 2.20 |
| Yang et al. [38] | 62.4 | 8.4 | 90 | 80 | 11.9* | 2.86 | 0.50 | 1.11 | 70~72 | - | <10 | 29.1 | 2.13 |
| Yang et al. [38] | 62.4 | 8.4 | 90 | 80 | 11.9* | 2.86 | 0.50 | 1.11 | 70~72 | - | <10 | 21.5 | 1.90 |
| Yang et al. [38] | 62.4 | 8.4 | 90 | 80 | 11.9* | 2.86 | 0.50 | 1.25 | 70~72 | - | <10 | 20.2 | 2.05 |
| Yang et al. [38] | 62.4 | 8.4 | 90 | 80 | 11.9* | 2.86 | 0.50 | 1.43 | 70~72 | - | <10 | 17.5 | 1.87 |
| Yang et al. [38] | 62.4 | 8.4 | 90 | 80 | 6.1* | 2.92 | 0.50 | 0.83 | 81~83 | - | <10 | 30.2 | 2.49 |
| Yang et al. [38] | 62.4 | 8.4 | 90 | 80 | 6.1* | 2.92 | 0.50 | 1.00 | 81~83 | - | <10 | 28.2 | 2.21 |
| Yang et al. [38] | 62.4 | 8.4 | 90 | 80 | 6.1* | 2.92 | 0.50 | 1.11 | 81~83 | - | <10 | 27.7 | 2.10 |
| Yang et al. [38] | 62.4 | 8.4 | 90 | 80 | 6.1* | 2.92 | 0.50 | 1.25 | 81~83 | - | <10 | 27.0 | 2.01 |
| Yang et al. [38] | 62.4 | 8.4 | 90 | 80 | 6.1* | 2.92 | 0.50 | 1.43 | 81~83 | - | <10 | 26.5 | 2.00 |
| Li et al. [39] | - | - | 7 | 80 | 6* | 2.68 | 0.50 | 0.67 | 104 | 30 | 50 | 15.2 | 1.44 |
| Li et al. [39] | - | - | 7 | 80 | 9* | 2.68 | 0.50 | 0.67 | 112 | 30 | 48 | 15.2 | 1.56 |
| Li et al. [39] | - | - | 7 | 80 | 12* | 2.68 | 0.50 | 0.67 | 114 | 30 | 50 | 16.4 | 1.59 |
| Li et al. [39] | - | - | 7 | 80 | 15* | 2.68 | 0.50 | 0.67 | 115 | 30 | 40 | 18.5 | 1.50 |
| Li et al. [39] | - | - | 7 | 80 | 18* | 2.68 | 0.50 | 0.67 | 117 | 30 | 44 | 16.2 | 1.62 |
| Li et al. [39] | - | - | 7 | 80 | 15* | 2.68 | 0.50 | 0.67 | 110 | 30 | 36 | 18.2 | 1.70 |
| Li et al. [39] | - | - | 7 | 80 | 15* | 2.68 | 0.50 | 0.67 | 108 | 30 | 49 | 15.8 | 1.58 |
| Li et al. [39] | - | - | 7 | 80 | 9* | 2.68 | 0.50 | - | 111 | 30 | 41 | 16.6 | 1.54 |
| Li et al. [39] | - | - | 7 | 80 | 6* | 2.68 | 0.50 | - | 112 | 30 | 50 | 17.2 | 1.58 |
| Li et al. [39] | - | - | 28 | 80 | 6* | 2.68 | 0.50 | 0.67 | 104 | 30 | 50 | 22.6 | 2.49 |
| Li et al. [39] | - | - | 28 | 80 | 9* | 2.68 | 0.50 | 0.67 | 112 | 30 | 48 | 26.2 | 2.33 |
| Li et al. [39] | - | - | 28 | 80 | 12* | 2.68 | 0.50 | 0.67 | 114 | 30 | 50 | 24.2 | 2.36 |
| Li et al. [39] | - | - | 28 | 80 | 15* | 2.68 | 0.50 | 0.67 | 115 | 30 | 40 | 27.3 | 2.75 |
| Li et al. [39] | - | - | 28 | 80 | 18* | 2.68 | 0.50 | 0.67 | 117 | 30 | 44 | 26.7 | 2.76 |
| Li et al. [39] | - | - | 28 | 80 | 15* | 2.68 | 0.50 | 0.67 | 110 | 30 | 36 | 26.5 | 2.48 |
| Li et al. [39] | - | - | 28 | 80 | 15* | 2.68 | 0.50 | 0.67 | 108 | 30 | 49 | 23.1 | 2.27 |
| Li et al. [39] | - | - | 28 | 80 | 9* | 2.68 | 0.50 | - | 111 | 30 | 41 | 28.2 | 2.97 |
| Li et al. [39] | - | - | 28 | 80 | 6* | 2.68 | 0.50 | - | 112 | 30 | 50 | 30.2 | 2.90 |
| Li et al. [39] | - | - | 90 | 80 | 6* | 2.68 | 0.50 | 0.67 | 104 | 30 | 50 | 29.9 | 3.54 |
| Li et al. [39] | - | - | 90 | 80 | 9* | 2.68 | 0.50 | 0.67 | 112 | 30 | 48 | 32.0 | 3.42 |
| Li et al. [39] | - | - | 90 | 80 | 12* | 2.68 | 0.50 | 0.67 | 114 | 30 | 50 | 33.0 | 3.18 |
| Li et al. [39] | - | - | 90 | 80 | 15* | 2.68 | 0.50 | 0.67 | 115 | 30 | 40 | 37.9 | 3.15 |
| Li et al. [39] | - | - | 90 | 80 | 18* | 2.68 | 0.50 | 0.67 | 117 | 30 | 44 | 37.1 | 3.21 |
| Li et al. [39] | - | - | 90 | 80 | 15* | 2.68 | 0.50 | 0.67 | 110 | 30 | 36 | 34.8 | 3.18 |
| Li et al. [39] | - | - | 90 | 80 | 15* | 2.68 | 0.50 | 0.67 | 108 | 30 | 49 | 34.2 | 2.69 |
| Li et al. [39] | - | - | 90 | 80 | 9* | 2.68 | 0.50 | - | 111 | 30 | 41 | 32.6 | 3.14 |
| Li et al. [39] | - | - | 90 | 80 | 6* | 2.68 | 0.50 | - | 112 | 30 | 50 | 39.6 | 3.11 |
| Xing [40] | 52.9 | 8.4 | 7 | 80 | 15* | 2.80 | 0.50 | 0.67 | 104 | 30 | 44 | 20.4 | 1.61 |
| Xing [40] | 52.9 | 8.4 | 7 | 80 | 15* | 2.80 | 0.50 | 0.67 | 108 | 30 | 49 | 19.9 | 1.57 |
| Xing [40] | 52.9 | 8.4 | 7 | 80 | 15* | 2.80 | 0.50 | 0.67 | 110 | 30 | 36 | 19.2 | 1.53 |
| Xing [40] | 52.9 | 8.4 | 7 | 80 | 15* | 2.80 | 0.50 | 0.67 | 114 | 30 | 45 | 18.4 | 1.50 |
| Xing [40] | 52.9 | 8.4 | 7 | 80 | 15* | 2.80 | 0.50 | 0.67 | 115 | 30 | 40 | 18.4 | 1.50 |
| Xing [40] | 52.9 | 8.4 | 28 | 80 | 15* | 2.80 | 0.50 | 0.67 | 104 | 30 | 44 | 29.7 | 3.04 |
| Xing [40] | 52.9 | 8.4 | 28 | 80 | 15* | 2.80 | 0.50 | 0.67 | 108 | 30 | 49 | 29.7 | 3.02 |
| Xing [40] | 52.9 | 8.4 | 28 | 80 | 15* | 2.80 | 0.50 | 0.67 | 110 | 30 | 36 | 28.5 | 2.91 |
| Xing [40] | 52.9 | 8.4 | 28 | 80 | 15* | 2.80 | 0.50 | 0.67 | 114 | 30 | 45 | 27.4 | 2.84 |
| Xing [40] | 52.9 | 8.4 | 28 | 80 | 15* | 2.80 | 0.50 | 0.67 | 115 | 30 | 40 | 27.1 | 2.75 |
| Xing [40] | 52.9 | 8.4 | 90 | 80 | 15* | 2.80 | 0.50 | 0.67 | 104 | 30 | 44 | 40.9 | 3.43 |
| Xing [40] | 52.9 | 8.4 | 90 | 80 | 15* | 2.80 | 0.50 | 0.67 | 108 | 30 | 49 | 40.2 | 3.37 |
| Xing [40] | 52.9 | 8.4 | 90 | 80 | 15* | 2.80 | 0.50 | 0.67 | 110 | 30 | 36 | 39.4 | 3.26 |
| Xing [40] | 52.9 | 8.4 | 90 | 80 | 15* | 2.80 | 0.50 | 0.67 | 114 | 30 | 45 | 38.3 | 3.19 |
| Xing [40] | 52.9 | 8.4 | 90 | 80 | 15* | 2.80 | 0.50 | 0.67 | 115 | 30 | 40 | 37.5 | 3.15 |
| C. Parra et al. [41] | 39.42 | - | 7 | 12 | 19.2 | - | 0.37 | 0.65 | 178.75 | 53 | - | 26.56^#^ | 1.92^※^ |
| C. Parra et al. [41] | 39.42 | - | 28 | 12 | 19.2 | - | 0.37 | 0.65 | 178.75 | 53 | - | 33.12^#^ | 2.34^※^ |
| C. Parra et al. [41] | 39.42 | - | 90 | 12 | 19.2 | - | 0.37 | 0.65 | 178.75 | 53 | - | 39.94^#^ | 3.32^※^ |
| C. Parra et al. [41] | 39.42 | - | 7 | 12 | 19.2 | - | 0.34 | 0.55 | 178.75 | 53 | - | 32.17^#^ | 2.67^※^ |
| C. Parra et al. [41] | 39.42 | - | 28 | 12 | 19.2 | - | 0.34 | 0.55 | 178.75 | 53 | - | 40.49^#^ | 3.03^※^ |
| C. Parra et al. [41] | 39.42 | - | 90 | 12 | 19.2 | - | 0.34 | 0.55 | 178.75 | 53 | - | 49.70^#^ | 3.68^※^ |
| C. Parra et al. [41] | 48.07 | - | 7 | 12 | 19.2 | - | 0.34 | 0.55 | 178.75 | 53 | - | 43.15^#^ | 2.90^※^ |
| C. Parra et al. [41] | 48.07 | - | 28 | 12 | 19.2 | - | 0.34 | 0.55 | 178.75 | 53 | - | 49.26^#^ | 3.12^※^ |
| C. Parra et al. [41] | 48.07 | - | 90 | 12 | 19.2 | - | 0.34 | 0.55 | 178.75 | 53 | - | 63.70^#^ | 4.10^※^ |
| C. Parra et al. [41] | 48.07 | - | 7 | 12 | 19.2 | - | 0.31 | 0.45 | 180 | 53 | - | 55.85^#^ | 3.85^※^ |
| C. Parra et al. [41] | 48.07 | - | 28 | 12 | 19.2 | - | 0.31 | 0.45 | 180 | 53 | - | 63.72^#^ | 3.86^※^ |
| C. Parra et al. [41] | 48.07 | - | 90 | 12 | 19.2 | - | 0.31 | 0.45 | 180 | 53 | - | 79.17^#^ | 4.19^※^ |
| C. Parra et al. [41] | 39.42 | - | 7 | 12 | 19.2 | - | 0.40 | 0.65 | 178.75 | 54 | 145 | 28.00^#^ | 2.22^※^ |
| C. Parra et al. [41] | 39.42 | - | 28 | 12 | 19.2 | - | 0.40 | 0.65 | 178.75 | 54 | 145 | 35.47^#^ | 3.34^※^ |
| C. Parra et al. [41] | 39.42 | - | 90 | 12 | 19.2 | - | 0.40 | 0.65 | 178.75 | 54 | 145 | 44.25^#^ | 3.47^※^ |
| C. Parra et al. [41] | 39.42 | - | 7 | 12 | 19.2 | - | 0.36 | 0.55 | 178.75 | 54 | 135 | 33.42^#^ | 2.31^※^ |
| C. Parra et al. [41] | 39.42 | - | 28 | 12 | 19.2 | - | 0.36 | 0.55 | 178.75 | 54 | 135 | 41.43^#^ | 3.52^※^ |
| C. Parra et al. [41] | 39.42 | - | 90 | 12 | 19.2 | - | 0.36 | 0.55 | 178.75 | 54 | 135 | 52.35^#^ | 3.98^※^ |
| C. Parra et al. [41] | 48.07 | - | 7 | 12 | 19.2 | - | 0.36 | 0.55 | 178.75 | 54 | 140 | 42.97^#^ | 3.61^※^ |
| C. Parra et al. [41] | 48.07 | - | 28 | 12 | 19.2 | - | 0.36 | 0.55 | 178.75 | 54 | 140 | 46.85^#^ | 4.25^※^ |
| C. Parra et al. [41] | 48.07 | - | 90 | 12 | 19.2 | - | 0.36 | 0.55 | 178.75 | 54 | 140 | 60.65^#^ | 4.59^※^ |
| C. Parra et al. [41] | 48.07 | - | 7 | 12 | 19.2 | - | 0.32 | 0.45 | 180 | 54 | 140 | 47.71^#^ | 3.75^※^ |
| C. Parra et al. [41] | 48.07 | - | 28 | 12 | 19.2 | - | 0.32 | 0.45 | 180 | 54 | 140 | 65.37^#^ | 4.69^※^ |
| C. Parra et al. [41] | 48.07 | - | 90 | 12 | 19.2 | - | 0.32 | 0.45 | 180 | 54 | 140 | 73.70^#^ | 4.76^※^ |

*the stone powder content in this paper are calculated as powder with particle size below 160μm.

^#^the compressive strength in calculated using formula *f*_cu_=1.22*f*_c_, where *f*_cu_ is cubic compressive strength, *f*_c_ is cylinder compressive strength.

^※^the splitting tensile strength were tested using cylinder specimens.
